# Oral Adverse Events Following COVID-19 Vaccination: Analysis of VAERS Reports

**DOI:** 10.3389/fpubh.2022.952781

**Published:** 2022-07-11

**Authors:** Abanoub Riad, Ave Põld, Elham Kateeb, Sameh Attia

**Affiliations:** ^1^Department of Public Health, Faculty of Medicine, Masaryk University, Brno, Czechia; ^2^Department of Oral, Dental and Maxillofacial Diseases, University Hospital Heidelberg, Heidelberg, Germany; ^3^Oral Health Research and Promotion Unit, Al-Quds University, Jerusalem, Palestine; ^4^Public Policy Center, The University of Iowa, Iowa City, IA, United States; ^5^Department of Oral and Maxillofacial Surgery, Justus-Liebig-University, Giessen, Germany

**Keywords:** anaphylaxis, COVID-19 vaccines, drug-related side effects and adverse reactions, oral manifestations, pharmacovigilance oral adverse events following COVID-19 vaccination 2

## Abstract

**Background:**

Oral adverse events (AEs) following COVID-19 vaccination have been sporadically reported during the previous months, warranting further investigation for their prevalence and suspected relationship with vaccine-elicited immune response.

**Methods:**

A retrospective analysis using the Vaccine Adverse Event Reporting System (VAERS) data was conducted to evaluate AEs within the oral cavity (mucosa, tongue, lips, palate, dentition, salivary glands) and AEs involving taste and other sensations. Oral AEs reported after receiving COVID-19 vaccination (test group) and seasonal influenza vaccination (control group) were extracted and cross-tabulated to assess their relative prevalence.

**Results:**

Among the 128 solicited (suspected) oral AEs, oral paresthesia (0.872%) was most reported after receiving COVID-19 vaccines, followed by the swelling of lips (0.844%), ageusia (0.722%), oral hypoesthesia (0.648%), swollen tongue (0.628%), and dysgeusia (0.617%). The reported prevalence of oral AEs was higher in the COVID-19 vaccine group than in the seasonal influenza group. The distribution pattern of the most reported oral AEs was similar for both COVID-19 and seasonal influenza vaccines. Female sex, older age (>39 years old), primer doses, and mRNA-based COVID-19 vaccines exhibited a higher reported prevalence of oral AEs.

**Conclusion:**

Within the limitations of this study, COVID-19 vaccines were found to be associated with rare oral AEs that are predominantly similar to those emerging following seasonal influenza vaccines. The most commonly reported oral AEs were oral paraesthesia (mouth-tingling), lip swelling, and ageusia, representing various pathophysiologic pathways that remain unclear. Taste-related AEs should be acknowledged in the context of the COVID-19 pandemic and the public should be adequately informed about a potential taste dysfunction after receiving the COVID-19 vaccination. Dentists and dental teams need to be aware of the prevalence, severity, and prognosis of oral AEs to inform their patients and increase public confidence in vaccines.

## Introduction

A wide array of clinical manifestations associated with coronavirus disease (COVID-19) have been reported within the oral cavity, including taste dysfunction, oral mucosal lesions, and salivary gland disorders (1). Therefore, dentists and dental team members encountered additional challenges in providing their services amid the pandemic while attempting to protect their patients and colleagues from cross-infection (2).

Fortunately, a strong global collaboration between pharmaceutical companies enabled the rapidly developing vaccines against this novel respiratory disease leading to certain vaccines receiving emergency authorization by the end of the first year of the pandemic. As vaccines offer the best solution to control this pandemic by establishing herd immunity, it is essential to achieve substantial vaccine uptake levels across the global community (3). To ensure a high vaccine uptake and prevent vaccine hesitancy, it is necessary to manage with its key triggers including the fear of potential post-vaccination side effects (3).

Individual reports were published sporadically during the previous months about oral adverse events (AEs) that emerged after receiving various COVID-19 vaccines, thus, warranting further investigation by epidemiologic researchers and careful attention by dental practitioners (4). The overarching aim of this study was to evaluate the oral AEs reported within the United States (US) population following COVID-19 vaccination, their prevalence and demographic risk factors, and compare them against oral AEs of seasonal influenza.

## Materials and Methods

### Design

A retrospective analysis for the Vaccine Adverse Event Reporting System (VAERS), an open-access database co-managed by the US Food and Drug Administration (FDA) and the Centers for Diseases Control and Prevention (CDC), was conducted in April 2022 (5). VAERS reports had been accessed through the CDC Wide-ranging Online Data for Epidemiologic Research (WONDER) tool, which provides summarized frequencies of reported symptoms based on the Medical Dictionary for Regulatory Activities (MedDRA) scheme (5, 6).

### Population

All VAERS reports of the individuals who received COVID-19 vaccination from January 1st to December 31st, 2021, were accessed through the WONDER tool and used as a “test group.” To select an appropriate “control group,” VAERS reports of all vaccines administered during 2021 were thoroughly examined. The decision to use seasonal influenza vaccines as a “control group” was made based on the following reasons: (a) seasonal influenza and COVID-19 vaccines are recommended/administered to all age groups and sexes in all US states and territories indiscriminately, (b) the frequency of seasonal influenza vaccine-related AEs reported during 2021 came second after the frequency of COVID-19 vaccine-related AEs, (c) both vaccines are primarily administered through intramuscular injection, and (d) both vaccines target respiratory infections that can spread similarly and synergistically in the community leading to similar clinical complications (7).

### Variables

**Figure 1:**
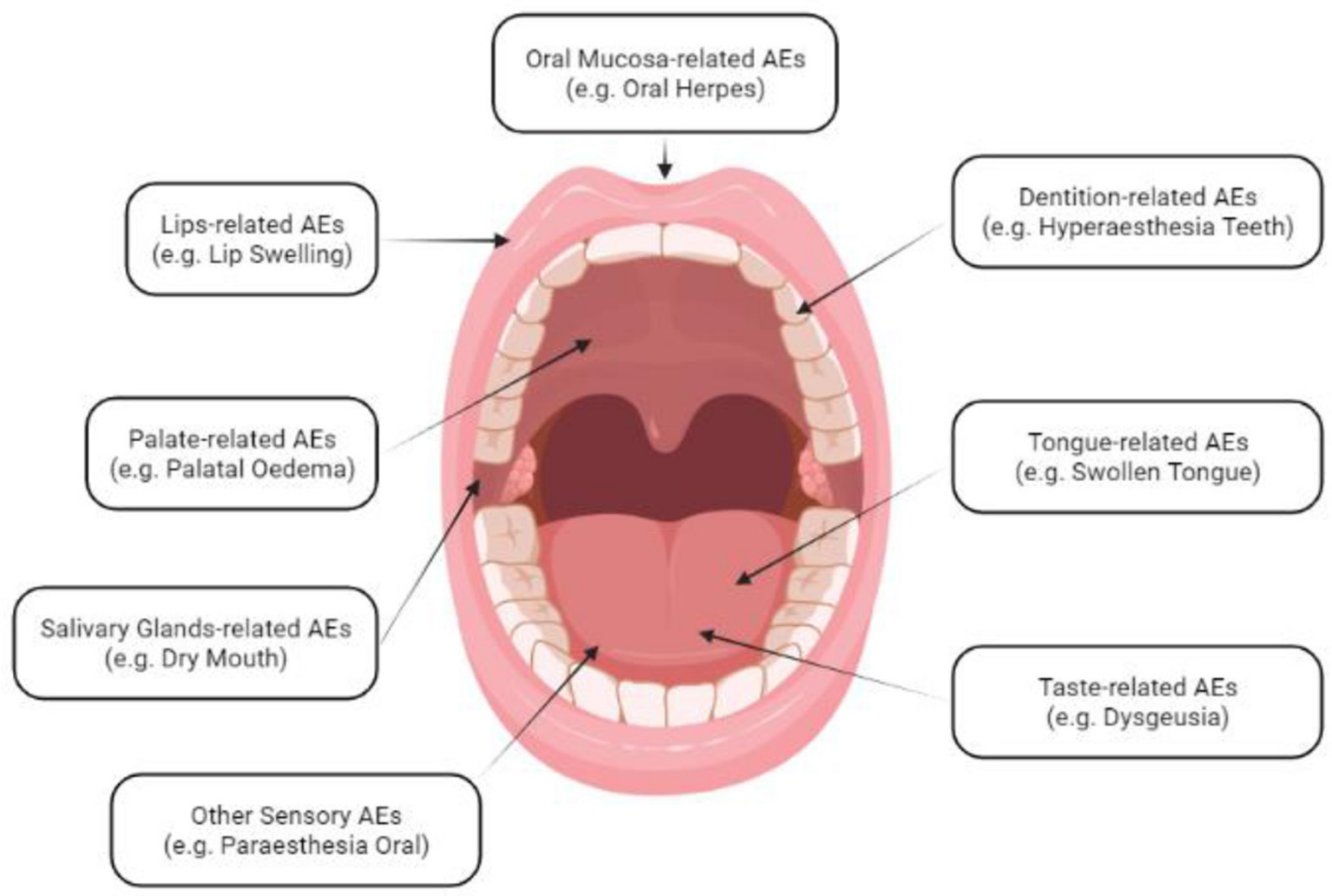
Anatomo-physiological Scheme of Oral Adverse Events Following Immunization (AEFI); created in BioRender.com.

MedDRA uses a logical classification hierarchy consisting of five levels starting from the “System Organ Class” level e.g., (gastrointestinal disorders) until the “Preferred Term” and the “Lowest Level Term” e.g., (aphthous stomatitis) and (aphthous ulcer), respectively (6). Oral AEs are scattered across various levels of the MedDRA hierarchy; therefore, we developed an anatomo-physiological scheme to extract all potential AEs related to oral cavity structures and functions. Our *de novo* scheme divided the oral cavity into six regions, including oral mucosa (e.g., oral herpes), tongue (e.g., swollen tongue), lips (e.g., lip swelling), palate (e.g., palatal oedema), salivary glands (e.g., dry mouth), and dentition (e.g., hyperaesthesia teeth), and two functions, including taste (e.g., dysgeusia) and other sensory disorders (e.g., oral paraesthesia) (Figure 1).

An exhaustive list of potential oral AEs (*n* = 310) was extracted based on our proposed scheme, and two oral surgery specialists reviewed it for further validation and filtration (Supplementary Table S1). A total of 182 preferred terms / lowest level terms (PT/LLT) had been excluded from the original list due to de-duplication (*n* = 43), being of congenital or developmental nature e.g., ankyloglossia congenital (*n* = 16), behavioral and traumatic injuries e.g., tooth fracture (*n* = 20), clinical dental procedures e.g., x-ray dental (*n* = 42), chronic conditions e.g., salivary gland cancer (*n* = 52) and irrelevant to oral cavity e.g., oral contraception (*n* = 9). A final list of 128 potential oral AEs was used in the downstream analyses.

### Analyses

The primary outcome was the proportion of oral AEs within all VAERS reports of the same vaccine group, e.g., [(number of ageusia reports related to COVID-19 vaccines)/(total reports related to COVID-19 vaccines)] ^*^ 100. The secondary outcome was the prevalence of reported AEs per 100,000 administered vaccine doses. Given the median age of the US population which is 38.5 years old, the age of 39 years was used as a cut-off point for the age-specific analysis of oral AEs prevalence (8). Chi-squared test (χ^2^) and Fisher's exact test were used to compare percentages and rates of oral AEs between COVID-19 vs. seasonal influenza vaccines, females vs. males, and ≤39 years old vs. >39 years old. Crosstabulation tests also compared oral AEs across various COVID-19 vaccine brands and doses. Moreover, taste-related AEs were compared between the pre-COVID-19 pandemic period (January 2010–December 2019) *vs*. the pandemic period (January 2020–December 2021). All analytical tests were performed using GraphPad Prism version 9.3.1 (GraphPad Software Inc. San Diego, CA, USA, 2021) and following the assumptions of confidence interval (CI) 95% and significance level (Sig.) ≤ 0.05.

## Results

### Demographic Characteristics

In total, 690,853 and 6,970 reports were received for COVID-19 and seasonal influenza vaccines, respectively, from individuals who were vaccinated in 2021, thus, constituting the denominator of the primary outcome in each vaccine group. Most reports in both COVID-19 (67.5%) and seasonal influenza (65.8%) groups were from females. The 65–79 years old was the most reporting age group in both COVID-19 (19.3%) and seasonal influenza (23.2%) groups, while the least reporting age group for COVID-19 vaccines was <6 years old, and for seasonal influenza vaccine was ≥80 years old (Table 1).

**Table 1:** Demographic characteristics of COVID-19 and seasonal influenza vaccines recipients in the United States, January–December 2021 (CDC; VAERS-WONDER).

**Variable**	**Outcome**	**COVID-19 vaccine** **(*n* = 507 M)**	**Seasonal influenza vaccine** **(*n* = 173.3 M)**	***Sig*** **.**
Received reports^†^	*N* (rate)	690,853 (136.3 per 100,000 doses)	6,970 (4.0 per 100,000 doses)	**<0.001**
^†^Sex	Female	466,323 (67.5%)	4,587 (65.8%)	**0.003**
	Male	211,597 (30.6%)	2,189 (31.4%)	0.162
	Unknown	12,933 (1.9%)	194 (2.8%)	**<0.001**
^†^Age group	<6 years	1,000 (0.1%)	439 (6.3%)	**<0.001**
	6–17 years	33,817 (4.9%)	734 (10.5%)	**<0.001**
	18–29 years	76,243 (11.0%)	625 (9.0%)	**<0.001**
	30–39 years	103,093 (14.9%)	789 (11.3%)	**<0.001**
	40–49 years	104,505 (15.1%)	655 (9.4%)	**<0.001**
	50–59 years	111,229 (16.1%)	877 (12.6%)	**<0.001**
	60–64 years	55,859 (8.1%)	596 (8.6%)	0.158
	65–79 years	133,504 (19.3%)	1,620 (23.2%)	**<0.001**
	80+ years	36,135 (5.2%)	370 (5.3%)	0.766
	Unknown	35,468 (5.1%)	265 (3.8%)	**<0.001**

*Chi-squared test (χ^2^) and Fisher's exact test had been used with a significance level (Sig.) < 0.05*.

*Bold values represents significant values*.

† *Total number of received reports*.

It is worth noting that the overall rate of reported AEs is significantly (*Sig*. < 0.001) higher in the COVID-19 group (136.3 reports per 100,000 administered doses) than in the seasonal influenza group (4 reports per 100,000 administered doses).

### Overall Prevalence of Oral AEs

In the domain of oral mucosa-related AEs, oral herpes was the most commonly reported AE in both COVID-19 and seasonal influenza vaccine groups (0.189 vs. 0.086%; *Sig*. = 0.050), followed by stomatitis and aphthous ulcer (0.168 vs. 0.158%; *Sig*. = 0.829), mouth swelling (0.131 vs. 0.115%; *Sig*. = 0.868), oral discomfort (0.108 vs. 0.072%; *Sig*. = 0.463), oral pain (0.103 vs. 0.086%; *Sig*. = 0.851), oral pruritus (0.065 vs. 0.014%; *Sig*. = 0.147), and oral mucosal blistering (0.057 vs. 0.043%; *Sig*. = 1.000). Despite the lack of statistically significant differences between vaccine groups in terms of the primary outcome (proportion of oral AEs to all reported AEs in VAERS), differences between vaccine groups in terms of the secondary outcome (proportion of oral AEs to all administered vaccine doses) were all statistically significant (*Sig*. < 0.001; Table 2).

**Table 2:** Oral adverse events reported after receiving COVID-19 and seasonal influenza vaccines in the United States, January–December 2021 (CDC; VAERS-WONDER).

**Preferred term**	**COVID-19 vaccine**		**Seasonal influenza vaccine**		***Sig***.		
	**% of Total AE** **(*n* = 690,853)**	**Rate per 100,000 doses**	**% of total AE** **(*n* = 6,970)**	**Rate per 100,000 doses**	**% of total AE**	**Rate per 100K D**	**Group**
Dental discomfort (10054217)	57 (0.008%)	0.011	1 (0.014%)	0.001	0.441	**0.006**	Dentition-Related AE^a^
Dental paraesthesia (10078276)	22 (0.003%)	0.004	0 (0%)	N/A	N/A	N/A	
Hyperaesthesia teeth (10082426)	159 (0.023%)	0.031	0 (0%)	N/A	N/A	N/A	
Hypoesthesia teeth (10051780)	15 (0.002%)	0.003	0 (0%)	N/A	N/A	N/A	
Toothache (10044055)	981 (0.142%)	0.193	3 (0.043%)	0.002	**0.023**	**<0.001**	
Ageusia (10001480)	4,985 (0.722%)	0.983	10 (0.143%)	0.006	**<0.001**	**<0.001**	Taste-Related AE
Dysgeusia (10013911)	4,263 (0.617%)	0.841	17 (0.244%)	0.010	**<0.001**	**<0.001**	
Hypogeusia (10020989)	141 (0.020%)	0.028	1 (0.014%)	0.001	1.000	**<0.001**	
Taste disorder (10082490)	2,193 (0.317%)	0.433	8 (0.115%)	0.005	**0.001**	**<0.001**	
Salivary duct stenosis (10039388)	1 (<0.001%)	<0.001	0 (0%)	N/A	N/A	N/A	Salivary glands and saliva-related AE^b^
Salivary gland pain (10039421)	19 (0.003%)	0.004	0 (0%)	N/A	N/A	N/A	
Salivary gland enlargement (10039408)	41 (0.006%)	0.008	0 (0%)	N/A	N/A	N/A	
Salivary gland calculus (10039394)	3 (<0.001%)	0.001	0 (0%)	N/A	N/A	N/A	
Salivary gland mass (10057002)	8 (0.001%)	0.002	0 (0%)	N/A	N/A	N/A	
Salivary gland disorder (10061935)	11 (0.002%)	0.002	0 (0%)	N/A	N/A	N/A	
Salivary hypersecretion (10039424)	259 (0.037%)	0.051	1 (0.014%)	0.001	0.529	**<0.001**	
Dry mouth (10013781)	2,080 (0.301%)	0.410	3 (0.043%)	0.002	**<0.001**	**<0.001**	
Aptyalism (10003068)	32 (0.005%)	0.006	0 (0%)	N/A	N/A	N/A	
Saliva discolouration (10049069)	4 (0.001%)	0.001	0 (0%)	N/A	N/A	N/A	
Saliva altered (10039379)	16 (0.002%)	0.003	1 (0.014%)	0.001	0.157	0.625	
Sialoadenitis (10040628)	31 (0.004%)	0.006	0 (0%)	N/A	N/A	N/A	
Non-infective sialoadenitis (10075243)	10 (0.001%)	0.002	0 (0%)	N/A	N/A	N/A	
Atrophic glossitis (10069085)	3 (<0.001%)	0.001	0 (0%)	N/A	N/A	N/A	Tongue-Related AE^c^
Hypertrophy of tongue papillae (10020893)	10 (0.001%)	0.002	0 (0%)	N/A	N/A	N/A	
Glossitis (10018386)	148 (0.021%)	0.029	0 (0%)	N/A	N/A	N/A	
Glossodynia (10018388)	564 (0.082%)	0.111	5 (0.072%)	0.003	0.773	**<0.001**	
Macroglossia (10025391)	2 (<0.001%)	<0.001	0 (0%)	N/A	N/A	N/A	
Plicated tongue (10035630)	9 (0.001%)	0.002	0 (0%)	N/A	N/A	N/A	
Swollen tongue (10042727)	4,340 (0.628%)	0.856	26 (0.373%)	0.015	**0.007**	**<0.001**	
Tongue oedema (10043967)	31 (0.004%)	0.006	0 (0%)	N/A	N/A	N/A	
Tongue blistering (10043942)	155 (0.022%)	0.031	3 (0.043%)	0.002	0.255	**<0.001**	
Tongue ulceration (10043991)	126 (0.018%)	0.025	0 (0%)	N/A	N/A	N/A	
Tongue coated (10043945)	39 (0.006%)	0.008	0 (0%)	N/A	N/A	N/A	
Tongue discolouration (10043949)	188 (0.027%)	0.037	1 (0.014%)	0.001	0.516	**<0.001**	
Trichoglossia (10080276)	7 (0.001%)	0.001	0 (0%)	N/A	N/A	N/A	
Tongue pigmentation (10069164)	4 (0.001%)	0.001	0 (0%)	N/A	N/A	N/A	
Tongue erythema (10079075)	138 (0.020%)	0.027	1 (0.014%)	0.001	0.740	**<0.001**	
Strawberry tongue (10051495)	4 (0.001%)	0.001	0 (0%)	N/A	N/A	N/A	
Tongue eruption (10052002)	66 (0.010%)	0.013	0 (0%)	N/A	N/A	N/A	
Tongue movement disturbance (10043963)	49 (0.007%)	0.010	0 (0%)	N/A	N/A	N/A	
Stiff tongue (10081491)	10 (0.001%)	0.002	0 (0%)	N/A	N/A	N/A	
Tongue paralysis (10043972)	24 (0.003%)	0.005	0 (0%)	N/A	N/A	N/A	
Tongue discomfort (10077855)	573 (0.083%)	0.113	0 (0%)	N/A	N/A	N/A	
Tongue disorder (10043951)	516 (0.075%)	0.102	4 (0.057%)	0.002	0.598	**<0.001**	
Tongue exfoliation (10064488)	12 (0.002%)	0.002	0 (0%)	N/A	N/A	N/A	
Tongue induration (10084548)	1 (<0.001%)	<0.001	0 (0%)	N/A	N/A	N/A	
Tongue dry (10049713)	75 (0.011%)	0.015	0 (0%)	N/A	N/A	N/A	
Tongue pruritus (10070072)	483 (0.070%)	0.095	4 (0.057%)	0.002	0.694	**<0.001**	
Tongue rough (10043977)	12 (0.002%)	0.002	0 (0%)	N/A	N/A	N/A	
Tongue spasm (10043981)	14 (0.002%)	0.003	0 (0%)	N/A	N/A	N/A	
Tongue fungal infection (10075845)	1 (<0.001%)	<0.001	0 (0%)	N/A	N/A	N/A	
Tongue thrust (10082545)	2 (<0.001%)	<0.001	0 (0%)	N/A	N/A	N/A	
Angular cheilitis (10002509)	14 (0.002%)	0.003	0 (0%)	N/A	N/A	N/A	Lip-Related AE^d^
Cheilitis (10008417)	303 (0.044%)	0.060	2 (0.029%)	0.001	0.547	**<0.001**	
Chapped lips (10049047)	210 (0.030%)	0.041	1 (0.014%)	0.001	0.443	**<0.001**	
Lip blister (10049307)	283 (0.041%)	0.056	4 (0.057%)	0.002	0.501	**<0.001**	
Lip discolouration (10024549)	102 (0.015%)	0.020	2 (0.029%)	0.001	0.343	**<0.001**	
Lip disorder (10048470)	125 (0.018%)	0.025	2 (0.029%)	0.001	0.514	**<0.001**	
Lip dry (10024552)	189 (0.027%)	0.037	3 (0.043%)	0.002	0.432	**<0.001**	
Lip erythema (10080124)	145 (0.021%)	0.029	1 (0.014%)	0.001	0.703	**<0.001**	
Lip exfoliation (10064482)	63 (0.009%)	0.012	2 (0.029%)	0.001	0.092	**0.003**	
Lip oedema (10024558)	60 (0.009%)	0.012	1 (0.014%)	0.001	0.615	**0.003**	
Lip pain (10024561)	266 (0.039%)	0.052	5 (0.072%)	0.003	0.161	**<0.001**	
Lip pruritus (10070721)	347 (0.050%)	0.068	2 (0.029%)	0.001	0.424	**<0.001**	
Lip scab (10082767)	7 (0.001%)	0.001	0 (0%)	N/A	N/A	N/A	
Lip swelling (10024570)	5,092 (0.737%)	1.000	41 (0.588%)	0.024	0.148	**<0.001**	
Lip ulceration (10024572)	24 (0.003%)	0.005	1 (0.014%)	0.001	0.131	0.219	
Palatal disorder (10052453)	18 (0.003%)	0.004	0 (0%)	N/A	N/A	N/A	Palate-Related AE^e^
Palatal oedema (10056998)	13 (0.002%)	0.003	0 (0%)	N/A	N/A	N/A	
Palatal swelling (10074403)	74 (0.011%)	0.015	2 (0.029%)	0.001	0.176	**0.001**	
Palatal ulcer (10077519)	5 (0.001%)	0.001	0 (0%)	N/A	N/A	N/A	
Anesthesia oral (10082548)	20 (0.003%)	0.004	0 (0%)	N/A	N/A	N/A	Other sensory AE
Paraesthesia oral (10057372)	6,024 (0.872%)	1.188	33 (0.473%)	0.019	**<0.001**	**<0.001**	
Hypoaesthesia oral (10057371)	4,477 (0.648%)	0.883	30 (0.430%)	0.017	**0.024**	**<0.001**	
Burn oral cavity (10075532)	9 (0.001%)	0.002	0 (0%)	N/A	N/A	N/A	
Burning mouth syndrome (10068065)	31 (0.004%)	0.006	0 (0%)	N/A	N/A	N/A	
Oral dysaesthesia (10050820)	4 (0.001%)	0.001	0 (0%)	N/A	N/A	N/A	
Aphthous ulcer (10002959)	340 (0.049%)	0.067	0 (0%)	N/A	N/A	N/A	Oral mucosa-related AE^f^
Circumoral oedema (10052250)	8 (0.001%)	0.002	0 (0%)	N/A	N/A	N/A	
Circumoral swelling (10081703)	44 (0.006%)	0.009	0 (0%)	N/A	N/A	N/A	
Coating in mouth (10075366)	21 (0.003%)	0.004	0 (0%)	N/A	N/A	N/A	
Leukoplakia oral (10024396)	5 (0.001%)	0.001	0 (0%)	N/A	N/A	N/A	
Mouth swelling (10075203)	908 (0.131%)	0.179	8 (0.115%)	0.005	0.868	**<0.001**	
Oedema mouth (10030110)	9 (0.001%)	0.002	1 (0.014%)	0.001	0.096	1.000	
Oral blood blister (10076590)	46 (0.007%)	0.009	1 (0.014%)	0.001	0.376	**0.022**	
Oral candidiasis (10030963)	108 (0.016%)	0.021	0 (0%)	N/A	N/A	N/A	
Oral discomfort (10030973)	747 (0.108%)	0.147	5 (0.072%)	0.003	0.463	**<0.001**	
Oral disorder (10067621)	191 (0.028%)	0.038	2 (0.029%)	0.001	0.720	**<0.001**	
Oral fungal infection (10061324)	14 (0.002%)	0.003	0 (0%)	N/A	N/A	N/A	
Oral herpes (10067152)	1,309 (0.189%)	0.258	6 (0.086%)	0.003	**0.050**	**<0.001**	
Oral lichen planus (10030983)	39 (0.006%)	0.008	1 (0.014%)	0.001	0.331	**0.039**	
Oral lichenoid reaction (10083833)	2 (<0.001%)	<0.001	0 (0%)	N/A	N/A	N/A	
Oral mucosa erosion (10064594)	1 (<0.001%)	<0.001	0 (0%)	N/A	N/A	N/A	
Oral Mucosal Blistering (10030995)	394 (0.057%)	0.078	3 (0.043%)	0.002	1.000	**<0.001**	
Oral mucosal discolouration (10030996)	6 (0.001%)	0.001	0 (0%)	N/A	N/A	N/A	
Oral mucosal eruption (10030997)	175 (0.025%)	0.035	4 (0.057%)	0.002	0.106	**<0.001**	
Oral mucosal erythema (10067418)	78 (0.011%)	0.015	0 (0%)	N/A	N/A	N/A	
Oral mucosal exfoliation (10064487)	32 (0.005%)	0.006	0 (0%)	N/A	N/A	N/A	
Oral mucosal roughening (10084009)	10 (0.001%)	0.002	0 (0%)	N/A	N/A	N/A	
Oral pain (10031009)	715 (0.103%)	0.141	6 (0.086%)	0.003	0.851	**<0.001**	
Oral pigmentation (10077552)	1 (<0.001%)	<0.001	0 (0%)	N/A	N/A	N/A	
Oral pruritus (10052894)	452 (0.065%)	0.089	1 (0.014%)	0.001	0.147	**<0.001**	
Oral purpura (10083533)	4 (0.001%)	0.001	0 (0%)	N/A	N/A	N/A	
Oral pustule (10056674)	12 (0.002%)	0.002	0 (0%)	N/A	N/A	N/A	
Oral viral infection (10065234)	2 (<0.001%)	<0.001	0 (0%)	N/A	N/A	N/A	
Oropharyngeal blistering (10067950)	46 (0.007%)	0.009	0 (0%)	N/A	N/A	N/A	
Oropharyngeal plaque (10067721)	6 (0.001%)	0.001	0 (0%)	N/A	N/A	N/A	
Perioral dermatitis (10034541)	8 (0.001%)	0.002	0 (0%)	N/A	N/A	N/A	
Stomatitis (10042128)	824 (0.119%)	0.163	11 (0.158%)	0.006	0.379	**<0.001**	

*Chi-squared test (χ^2^) and Fisher's exact test had been used with a significance level (Sig.) < 0.05*.

a *The preferred term Sensitivity of Teeth (10040012) was not reported in any vaccine groups*.

b *The preferred terms Salivary Duct Obstruction (10039386), Salivary Duct Inflammation (10056681) and Salivary Gland Induration (10071363) were not reported in any vaccine groups*.

c *The preferred terms Acquired Macroglossia (10058835), Ankyloglossia Acquired (10049243), Atrophy of Tongue Papillae (10003712), and Tongue Black Hairy (10043941) were not reported in any vaccine groups*.

d *The preferred term Lip Erosion (10051992) was not reported in any vaccine groups*.

e *The preferred term Palatal Palsy (10072012) was not reported in any vaccine groups*.

f *The preferred terms Aphthous Stomatitis (10002958), Buccal Mucosal Roughening (10048479), Mouth Plaque (10028032), Mouth Ulceration (10028034), Oral Soft Tissue Disorder (10061326), Oral Mucosal Hypertrophy (10062956), Oral Mucosal Petechiae (10030998), Oral Mucosal Scab (10082769), and Oral Papule (10031010) were not reported in any vaccine groups*.

*Bold values represents significant values*.

Swollen tongue (0.628% *vs*. 0.072%; *Sig*. = 0.007) was the most common tongue-related AE in both COVID-19 and seasonal influenza groups, followed by tongue discomfort (0.083 vs. 0%), tongue disorder (0.075 vs. 0.057%; *Sig*. = 0.598), and tongue pruritus (0.070 vs. 0.057%; *Sig*. = 0.694). Lip swelling (0.737 vs. 0.588%; *Sig*. = 0.148) and lip pruritus (0.050 vs. 0.029%; *Sig*. = 0.424) were the most common lip-related AEs in both vaccine groups. Dry mouth (0.301 vs. 0.043%; *Sig*. < 0.001) was the most common salivary glands-related AE, while toothache (0.142 vs. 0.043%; *Sig*. = 0.023) was the most common dentition-related AE in both vaccine groups (Supplementary Figure S2).

**Figure 2:**
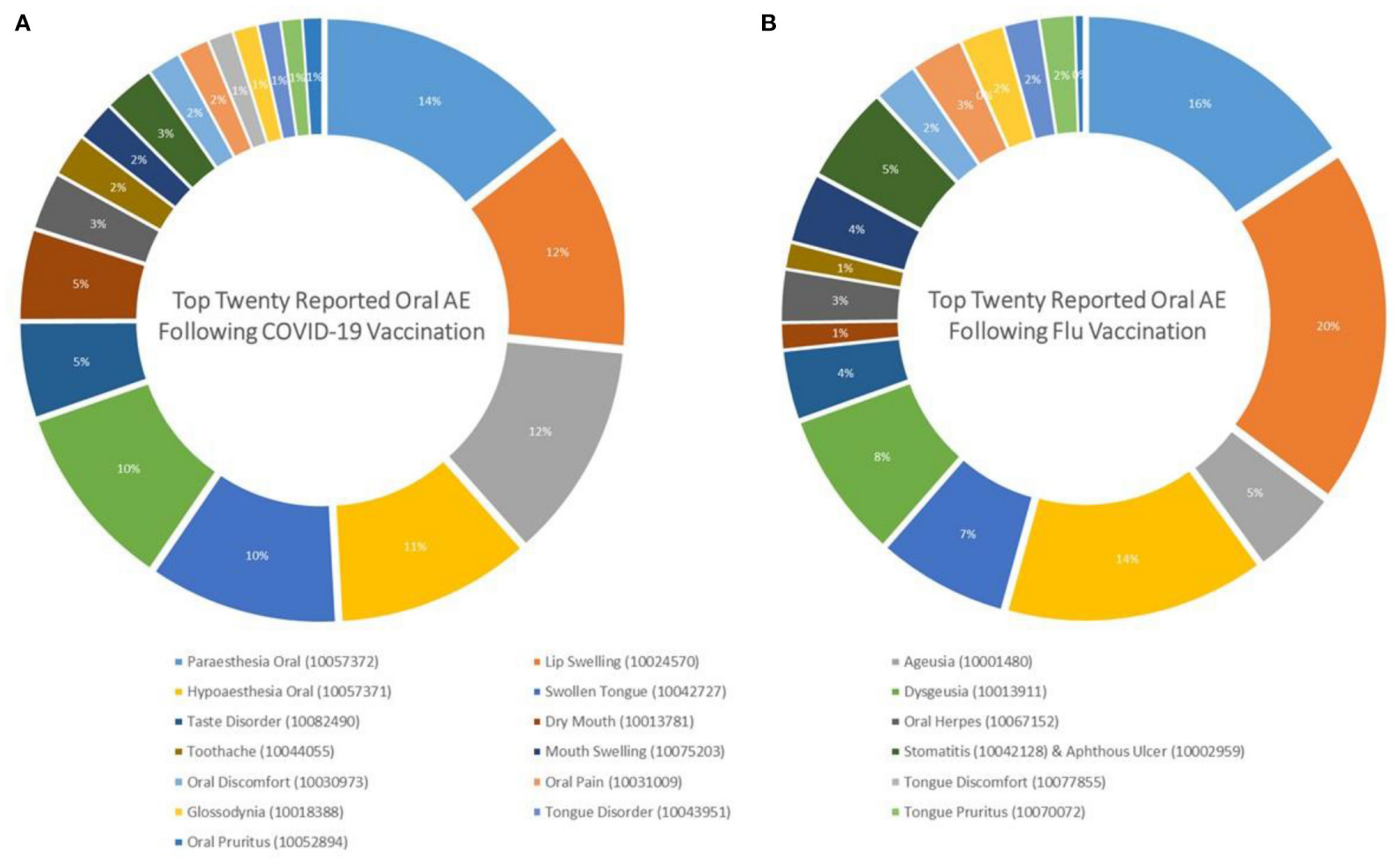
Top twenty oral adverse events reported after **(A)** COVID-19 and **(B)** Seasonal Influenza Vaccination in the United States, January–December 2021 (CDC; VAERS-WONDER).

Ageusia (0.722 vs. 0.143%; *Sig*. < 0.001) was the most common taste-related AE in both vaccine groups, followed by dysgeusia (0.617 vs. 0.244%; *Sig*. < 0.001) and taste disorder (0.317 vs. 0.115%; *Sig*. < 0.001). Oral paraesthesia (0.872 vs. 0.473%; *Sig*. < 0.001) and oral hypoaesthesia (0.648 vs. 0.430%; *Sig*. < 0.001) were the most frequently reported sensory AEs in both vaccine groups (Figure 2).

### Sex- and Age-Specific Prevalence

**Figure 3:**
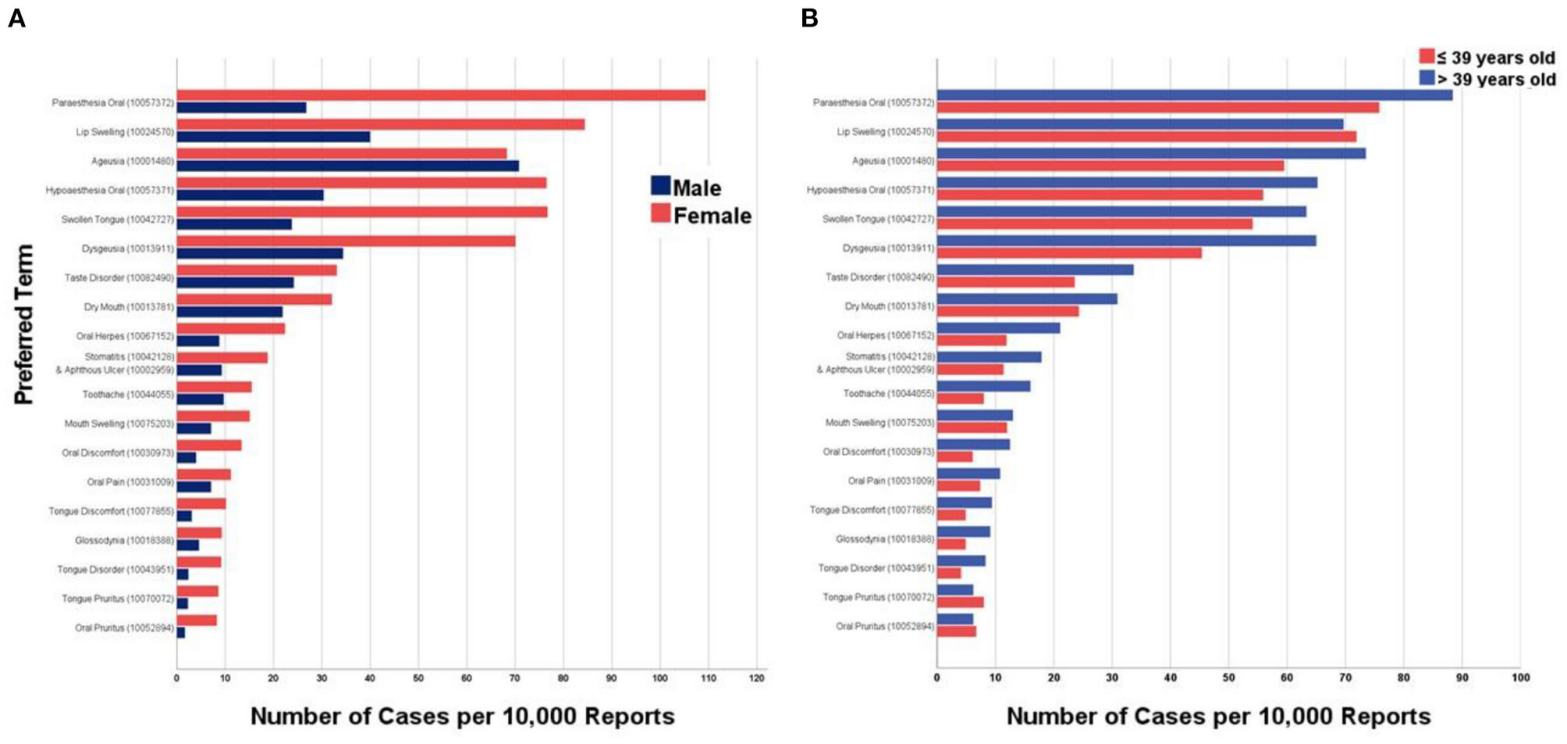
Top twenty oral adverse events reported after COVID-19 vaccination in the United States stratified by **(A)** Sex and **(B)** Age Group, January–December 2021 (CDC; VAERS-WONDER).

In the COVID-19 group, analysis of the top twenty oral AEs sex-specific prevalence revealed that females had a significantly (*Sig*. < 0.001) higher prevalence than males in all solicited AEs except for ageusia. The prevalence of ageusia was similar among females (0.683%) and males (0.708%), *Sig*. = 0.238. Likewise, females reported more oral AEs than males in the seasonal influenza group except for oral herpes, toothache, and tongue discomfort which were almost equally prevalent across sexes (Figure 3).

Interestingly, in the COVID-19 group, the older age group (>39 years old) had significantly (*Sig*. < 0.001) higher prevalence of oral AEs compared to the younger age group (≤39 years old) except for lip swelling, tongue pruritus, and oral pruritus. Similarly, the older age group reported oral AEs more frequently than the younger age group following seasonal influenza vaccination, except for dysgeusia, oral herpes and tongue pruritus (Table 3).

**Table 3:** Top twenty oral adverse events reported after COVID-19 and seasonal influenza vaccines in the United States stratified by sex and age group, January–December 2021 (CDC; VAERS-WONDER).

**#**	**Preferred term**	**COVID-19**			**Seasonal flu**		
		**Female** **(*n* = 489,571)**	**Male** **(*n* = 226,792)**	***Sig*** **.**	**Female** **(*n* = 4,629)**	**Male** **(*n* = 2,210)**	***Sig*** **.**
1	Paraesthesia oral (10057372)	5,355 (1.094%)	608 (0.268%)	**<0.001**	26 (0.562%)	7 (0.317%)	0.195
2	Lip swelling (10024570)	4,132 (0.844%)	907 (0.400%)	**<0.001**	35 (0.756%)	6 (0.271%)	**0.018**
3	Ageusia (10001480)	3,342 (0.683%)	1,605 (0.708%)	0.238	8 (0.173%)	2 (0.090%)	0.516
4	Hypoaesthesia oral (10057371)	3,743 (0.765%)	689 (0.304%)	**<0.001**	25 (0.540%)	5 (0.226%)	0.078
5	Swollen tongue (10042727)	3,749 (0.767%)	540 (0.238%)	**<0.001**	23 (0.497%)	3 (0.136%)	**0.021**
6	Dysgeusia (10013911)	3,431 (0.701%)	781 (0.344%)	**<0.001**	16 (0.346%)	1 (0.045%)	**0.018**
7	Taste disorder (10082490)	1,619 (0.331%)	549 (0.242%)	**<0.001**	6 (0.130%)	2 (0.090%)	1.000
8	Dry mouth (10013781)	1,573 (0.321%)	497 (0.219%)	**<0.001**	3 (0.065%)	0 (0%)	N/A
9	Oral herpes (10067152)	1,097 (0.224%)	200 (0.088%)	**<0.001**	4 (0.086%)	2 (0.090%)	1.000
10 & 11	Stomatitis (10042128) & Aphthous ulcer (10002959)	919 (0.188%)	211 (0.093%)	**<0.001**	8 (0.173%)	3 (0.136%)	1.000
12	Toothache (10044055)	758 (0.155%)	219 (0.097%)	**<0.001**	1 (0.022%)	2 (0.090%)	0.246
13	Mouth swelling (10075203)	738 (0.151%)	162 (0.071%)	**<0.001**	6 (0.130%)	2 (0.090%)	1.000
14	Oral discomfort (10030973)	654 (0.134%)	91 (0.040%)	**<0.001**	5 (0.108%)	0 (0%)	N/A
15	Oral pain (10031009)	547 (0.112%)	161 (0.071%)	**<0.001**	6 (0.130%)	0 (0%)	N/A
16	Tongue discomfort (10077855)	500 (0.102%)	71 (0.031%)	**<0.001**	0 (0%)	0 (0%)	N/A
17	Glossodynia (10018388)	457 (0.093%)	104 (0.046%)	**<0.001**	5 (0.108%)	0 (0%)	N/A
18	Tongue disorder (10043951)	448 (0.092%)	55 (0.024%)	**<0.001**	3 (0.065%)	1 (0.045%)	1.000
19	Tongue pruritus (10070072)	422 (0.086%)	53 (0.023%)	**<0.001**	4 (0.086%)	0 (0%)	N/A
20	Oral pruritus (10052894)	406 (0.083%)	39 (0.017%)	**<0.001**	1 (0.022%)	0 (0%)	N/A
**#**	**Preferred Term**	**COVID-19**			**Seasonal flu**		
		**≤39 years old** **(*****n*** **=** **223,053)**	**>39 years old** **(*****n*** **=** **471,468)**	***Sig***.	**≤39 years old** **(*****n*** **=** **2,606)**	**>39 years old** **(*****n*** **=** **4,099)**	***Sig***.
1	Paraesthesia oral (10057372)	1,691 (0.758%)	4,168 (0.884%)	**<0.001**	10 (0.384%)	23 (0.561%)	0.373
2	Lip swelling (10024570)	1,604 (0.719%)	3,288 (0.697%)	0.312	13 (0.499%)	27 (0.659%)	0.516
3	Ageusia (10001480)	1,327 (0.595%)	3,467 (0.735%)	**<0.001**	1 (0.038%)	10 (0.244%)	0.060
4	Hypoaesthesia oral (10057371)	1,246 (0.559%)	3,075 (0.652%)	**<0.001**	8 (0.307%)	23 (0.561%)	0.144
5	Swollen tongue (10042727)	1,206 (0.541%)	2,984 (0.633%)	**<0.001**	6 (0.230%)	18 (0.439%)	0.209
6	Dysgeusia (10013911)	1,013 (0.454%)	3,063 (0.650%)	**<0.001**	8 (0.307%)	9 (0.220%)	0.619
7	Taste disorder (10082490)	526 (0.236%)	1,589 (0.337%)	**<0.001**	2 (0.077%)	6 (0.146%)	0.496
8	Dry mouth (10013781)	542 (0.243%)	1,458 (0.309%)	**<0.001**	1 (0.038%)	2 (0.049%)	1.000
9	Oral herpes (10067152)	265 (0.119%)	996 (0.211%)	**<0.001**	3 (0.115%)	3 (0.073%)	0.683
10 & 11	Stomatitis (10042128) & Aphthous ulcer (10002959)	255 (0.114%)	845 (0.179%)	**<0.001**	2 (0.077%)	9 (0.220%)	0.220
12	Toothache (10044055)	178 (0.080%)	755 (0.160%)	**<0.001**	2 (0.077%)	1 (0.024%)	0.564
13	Mouth swelling (10075203)	268 (0.120%)	614 (0.130%)	0.271	2 (0.077%)	5 (0.122%)	0.713
14	Oral discomfort (10030973)	137 (0.061%)	587 (0.125%)	**<0.001**	1 (0.038%)	4 (0.098%)	0.655
15	Oral pain (10031009)	165 (0.074%)	510 (0.108%)	**<0.001**	0 (0%)	6 (0.146%)	N/A
16	Tongue discomfort (10077855)	109 (0.049%)	443 (0.094%)	**<0.001**	0 (0%)	0 (0%)	N/A
17	Glossodynia (10018388)	110 (0.049%)	428 (0.091%)	**<0.001**	0 (%)	2 (0.049%)	N/A
18	Tongue disorder (10043951)	91 (0.041%)	392 (0.083%)	**<0.001**	0 (0%)	4 (0.098%)	N/A
19	Tongue pruritus (10070072)	179 (0.080%)	292 (0.062%)	**0.006**	2 (0.077%)	2 (0.049%)	0.645
20	Oral pruritus (10052894)	149 (0.067%)	293 (0.062%)	0.473	0 (0%)	1 (0.024%)	N/A

*Chi-squared test (χ^2^) and Fisher's exact test had been used with a significance level (Sig.) < 0.05*.

*Bold values represents significant values*.

### Dose- and Vaccine Brand-Related Prevalence

**Figure 4:**
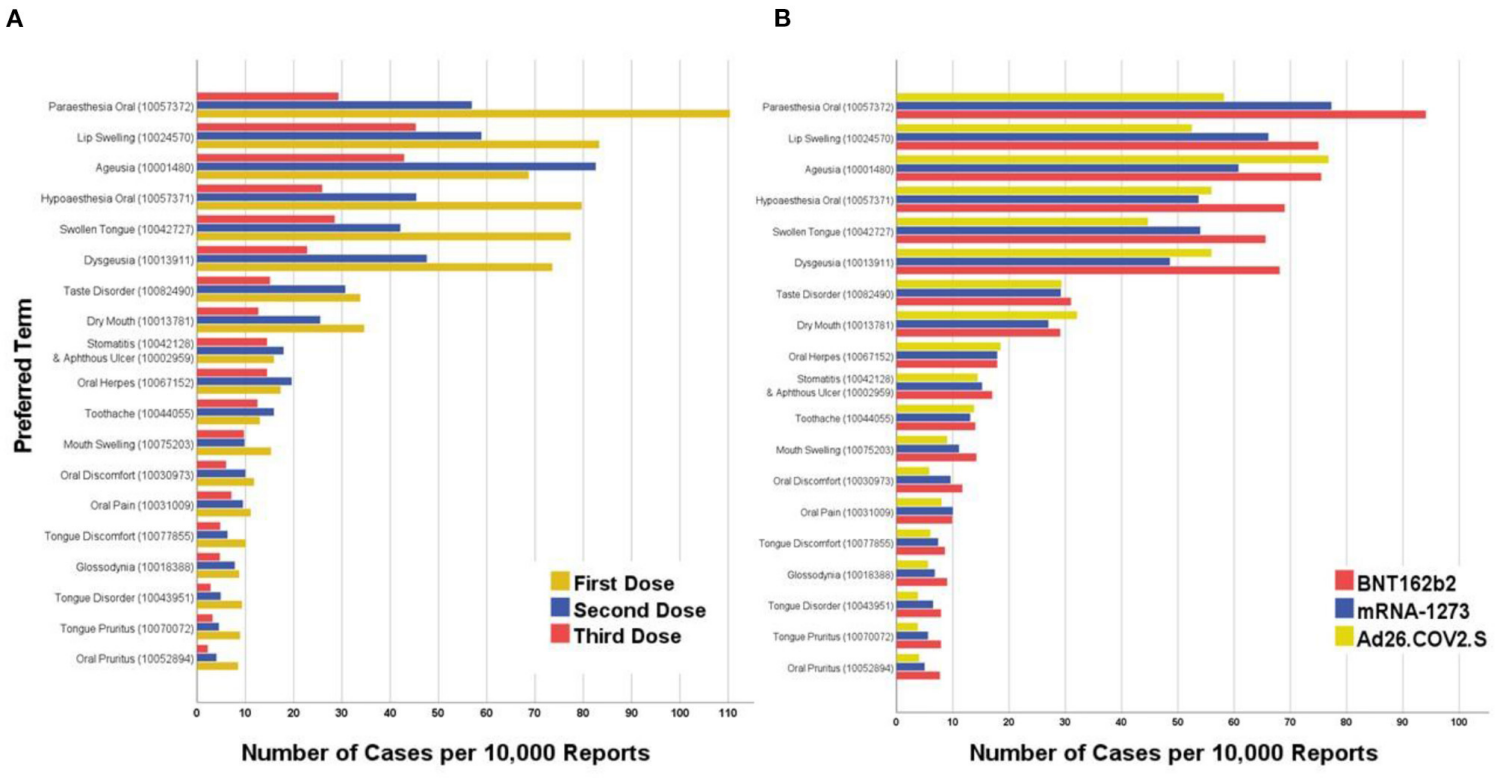
Top twenty oral adverse events reported after COVID-19 vaccination in the United States stratified by **(A)** Dose and **(B)** Vaccine Brand, January–December 2021 (CDC; VAERS-WONDER).

The first dose of COVID-19 vaccination was significantly (*Sig*. < 0.001) associated with more oral AEs than the second dose, except for ageusia, stomatitis and aphthous ulcer, oral herpes, and toothache where the second dose was associated more frequent oral AEs. On comparing the primer doses (first dose and second dose) with the booster doses (third dose), the primer doses were significantly associated with a higher prevalence of all solicited AEs compared to the booster doses (*Sig*. < 0.001; Figure 4).

Pfizer-BioNTech COVID-19 vaccine (BNT162b2) was associated with more frequently reported oral AEs than Moderna COVID-19 vaccine (mRNA-1273), except for oral herpes and oral pain. Overall, mRNA-based vaccines (BNT162b2 and mRNA-1273) had a significantly higher prevalence of oral paraesthesia (0.838 vs. 0.582%; *Sig*. < 0.001), lip swelling (0.706 vs. 0.525%; *Sig*. < 0.001), swollen tongue (0.599 vs. 0.447%; *Sig*. < 0.001), mouth swelling (0.127 vs. 0.090%; *Sig*. = 0.021), oral discomfort (0.106 vs. 0.058%; *Sig*. = 0.001), tongue disorder (0.072 vs. 0.038%; *Sig*. = 0.004), tongue pruritus (0.068 vs. 0.038%; *Sig*. = 0.011), and oral pruritus (0.063 vs. 0.040%; *Sig*. = 0.040; Tables 4, 5).

**Table 4:** Top twenty oral adverse events reported after COVID-19 vaccines in the United States stratified by dose, January–December 2021 (CDC; VAERS-WONDER).

**Order**	**Preferred term**	**First dose** **(*n* = 350,247)**	**Second dose** **(*n* = 227,377)**	**Third dose** **(*n* = 53,620)**	***Sig***.	
					**1st vs. 2nd**	**(1st & 2nd) vs. 3rd**
1	Paraesthesia oral (10057372)	3,868 (1.104%)	1,294 (0.569%)	157 (0.293%)	**<0.001**	**<0.001**
2	Lip swelling (10024570)	2,918 (0.833%)	1,339 (0.589%)	243 (0.453%)	**<0.001**	**<0.001**
3	Ageusia (10001480)	2,406 (0.687%)	1,877 (0.826%)	230 (0.429%)	**<0.001**	**<0.001**
4	Hypoaesthesia oral (10057371)	2,793 (0.797%)	1,033 (0.454%)	139 (0.259%)	**<0.001**	**<0.001**
5	Swollen tongue (10042727)	2,710 (0.774%)	958 (0.421%)	153 (0.285%)	**<0.001**	**<0.001**
6	Dysgeusia (10013911)	2,577 (0.736%)	1,082 (0.476%)	122 (0.228%)	**<0.001**	**<0.001**
7	Taste disorder (10082490)	1,183 (0.338%)	697 (0.307%)	81 (0.151%)	**0.042**	**<0.001**
8	Dry mouth (10013781)	1,212 (0.346%)	580 (0.255%)	68 (0.127%)	**<0.001**	**<0.001**
9	Oral herpes (10067152)	606 (0.173%)	445 (0.196%)	78 (0.145%)	**0.050**	0.061
10 & 11	Stomatitis (10042128) & Aphthous ulcer (10002959)	557 (0.159%)	406 (0.179%)	78 (0.145%)	0.080	0.266
12	Toothache (10044055)	455 (0.130%)	361 (0.159%)	67 (0.125%)	**0.005**	0.365
13	Mouth swelling (10075203)	536 (0.153%)	222 (0.098%)	52 (0.097%)	**<0.001**	**0.032**
14	Oral discomfort (10030973)	413 (0.118%)	227 (0.100%)	32 (0.060%)	**0.047**	**<0.001**
15	Oral pain (10031009)	389 (0.111%)	217 (0.095%)	38 (0.071%)	0.074	**0.016**
16	Tongue discomfort (10077855)	355 (0.101%)	144 (0.063%)	26 (0.048%)	**<0.001**	**0.003**
17	Glossodynia (10018388)	304 (0.087%)	178 (0.078%)	25 (0.047%)	0.284	**0.003**
18	Tongue disorder (10043951)	324 (0.093%)	112 (0.049%)	15 (0.028%)	**<0.001**	**<0.001**
19	Tongue pruritus (10070072)	313 (0.089%)	103 (0.045%)	17 (0.032%)	**<0.001**	**<0.001**
20	Oral pruritus (10052894)	299 (0.085%)	91 (0.040%)	12 (0.022%)	**<0.001**	**<0.001**

*Chi-squared test (χ^2^) and Fisher's exact test had been used with a significance level (Sig.) <0.05*.

*Bold values represents significant values*.

**Table 5:** Top twenty oral adverse events reported after COVID-19 vaccines in the United States stratified by vaccine type, January–December 2021 (CDC; VAERS-WONDER).

**Order**	**Preferred term**	**BNT162b2** **(*n* = 341,389)**	**mRNA-1273** **(*n* = 337,518)**	**Ad26.COV2.S** **(*n* = 50,138)**	***Sig***.	
					**Pfizer vs. Moderna**	**mRNA vs. Vector**
1	Paraesthesia oral (10057372)	3,212 (0.941%)	2,475 (0.773%)	292 (0.582%)	**<0.001**	**<0.001**
2	Lip swelling (10024570)	2,562 (0.750%)	2,232 (0.661%)	263 (0.525%)	**<0.001**	**<0.001**
3	Ageusia (10001480)	2,578 (0.755%)	2,053 (0.608%)	385 (0.768%)	**<0.001**	**0.027**
4	Hypoaesthesia oral (10057371)	2,354 (0.690%)	1,812 (0.537%)	281 (0.560%)	**<0.001**	0.145
5	Swollen tongue (10042727)	2,241 (0.656%)	1,823 (0.540%)	224 (0.447%)	**<0.001**	**<0.001**
6	Dysgeusia (10013911)	2,324 (0.681%)	1,639 (0.486%)	281 (0.560%)	**<0.001**	0.523
7	Taste disorder (10082490)	1,057 (0.310%)	986 (0.292%)	147 (0.293%)	0.191	0.800
8	Dry mouth (10013781)	992 (0.291%)	912 (0.270%)	161 (0.321%)	0.113	0.098
9	Oral herpes (10067152)	611 (0.179%)	603 (0.179%)	93 (0.185%)	0.977	0.702
10 & 11	Stomatitis (10042128) & Aphthous ulcer (10002959)	580 (0.170%)	512 (0.152%)	72 (0.144%)	0.065	0.385
12	Toothache (10044055)	477 (0.140%)	442 (0.131%)	69 (0.138%)	0.338	0.900
13	Mouth swelling (10075203)	485 (0.142%)	375 (0.111%)	45 (0.090%)	**<0.001**	**0.021**
14	Oral discomfort (10030973)	399 (0.117%)	324 (0.096%)	29 (0.058%)	**0.009**	**0.001**
15	Oral pain (10031009)	338 (0.099%)	336 (0.100%)	40 (0.080%)	0.969	0.208
16	Tongue discomfort (10077855)	295 (0.086%)	249 (0.074%)	30 (0.060%)	0.072	0.137
17	Glossodynia (10018388)	306 (0.090%)	229 (0.068%)	28 (0.056%)	**0.002**	0.080
18	Tongue disorder (10043951)	270 (0.079%)	219 (0.065%)	19 (0.038%)	**0.030**	**0.004**
19	Tongue pruritus (10070072)	271 (0.079%)	188 (0.056%)	19 (0.038%)	**<0.001**	**0.011**
20	Oral pruritus (10052894)	262 (0.077%)	169 (0.050%)	20 (0.040%)	**<0.001**	**0.040**

*Chi-squared test (χ^2^) and Fisher's exact test had been used with a significance level (Sig.) <0.05*.

*Bold values represents significant values*.

### Longitudinal Analysis of Taste-Related AEs

On comparing the overall percentage of taste-related AEs before (January 2010–December 2019) and during (January 2020–December 2021) the COVID-19 pandemic, ageusia was significantly more commonly reported during the pandemic (0.622 vs. 0.035%; *Sig*. < 0.001) than before the pandemic interval. Likewise, dysgeusia (0.581 vs. 0.182%; *Sig*. < 0.001), taste disorder (0.279 vs. 0.006%; *Sig*. < 0.001), and hypogeusia (0.019 vs. 0.006%; *Sig*. < 0.001) were significantly more common during the pandemic interval (Table 6).

**Table 6:** Taste-related adverse events reported after all vaccines in the United States, January 2010–December 2021 (CDC; VAERS-WONDER).

**Preferred term**	**Before COVID-19 pandemic; January 2010–December 2019** **(*n* = 311,941)**	**During COVID-19 Pandemic; January 2020–December 2021** **(*n* = 786,047)**	***Sig*** **.**
Ageusia (10001480)	109 (0.035%)	4,893 (0.622%)	**<0.001**
Dysgeusia (10013911)	567 (0.182%)	4,566 (0.581%)	**<0.001**
Taste disorder (10082490)	20 (0.006%)	2,193 (0.279%)	**<0.001**
Hypogeusia (10020989)	18 (0.006%)	146 (0.019%)	**<0.001**

*Fisher's exact test had been used with a significance level (Sig.) < 0.05*.

*Bold values represents significant values*.

## Discussion

The present analysis aimed to synthesize population-based evidence about oral adverse events (AEs) after COVID-19 vaccination utilizing a national surveillance database. Comparison between COVID-19 and seasonal influenza vaccines revealed a remarkable similarity in the distribution of the most reported oral AEs. Among the top twenty oral AEs, oral paresthesia was most reported after COVID-19 and seasonal influenza vaccination (14 and 16%, respectively), followed by lip swelling (12 and 20%), ageusia (12 and 5%), oral hypoesthesia (11 and 14%), swollen tongue (10 and 7%), dysgeusia (10 and 8%), taste disorder (5 and 4%), dry mouth (5 and 1%), and oral herpes (3 and 3%). Cohen et al. found that constitutional AEs such as headache, fatigue, and pyrexia had a significantly higher prevalence following COVID-19 than seasonal influenza and hepatitis B vaccines in the VAERS database (9). Likewise, a recent comprehensive analysis of the US and European Union surveillance systems indicated that the largest absolute risks following COVID-19 vaccines were constitutional, dermatological, neurological and gastrointestinal AEs (10). Given the nature of passive surveillance systems, including VAERS, which are inclined toward under-reporting than over-reporting, the higher prevalence of oral AEs following COVID-19 vaccines as compared with seasonal influenza vaccines can be attributed to reporting bias that might be triggered by public anxiety due to the novelty of COVID-19 vaccines (10, 11).

Taste disorders, including complete (ageusia) and partial (hypogeusia) loss of taste and disturbed taste (dysgeusia) had been among the most reported oral AEs following COVID-19 vaccination per our analysis. Taste dysfunction was depicted as one of the characteristic symptoms of COVID-19 infection due to its high prevalence, estimated to be 39.2% (CI 95%: 35.34–43.12%) according to latest meta-analyses (12–14). Therefore, public health authorities had broadly used taste dysfunction in case finding protocols and triage recommendations (15). Lechien et al. reported a series of six cases with new-onset taste disorders following COVID-19 vaccination with repeated negative PCR results for SARS-CoV-2. Thus ruling out the possibility of COVID-19 infection as the etiology of taste disorders in this particular case series; however it does not rule it out in the VAERS reports (16). On reviewing random VAERS reports manually, we found out that breakthrough infection was commonly reported in association with taste disorders. One of the hypotheses we suggest for the remarkably higher prevalence of ageusia (0.722 vs. 0.143%; *Sig*. < 0.001), dysgeusia (0.617 vs. 0.244%; *Sig*. < 0.001), and taste disorder (0.317 vs. 0.115%; *Sig*. < 0.001) among COVID-19 vaccinees is the increased public awareness of taste dysfunction as one of the characteristic symptoms of COVID-19 infection (17, 18). Our hypothesis can be supported by the overall reporting prevalence of taste disorders during the COVID-19 pandemic period compared to the pre-pandemic years.

Xerostomia, or dry mouth, was among the common oral AEs following COVID-19 vaccination with female and older age predominance and without a preference for a particular vaccine brand. Active surveillance through the cross-sectional studies of self-reported COVID-19 vaccines side effects revealed varying prevalence of xerostomia that ranged between 0.4 and 2.7% (19–21). It remains unclear whether the humoral immune response triggered by COVID-19 vaccination and manifested in salivary secretions has any link with salivary gland-AEs, including xerostomia (22, 23).

Anaphylactic symptoms e.g., lip swelling (MedDRA ID: 10024570), swollen tongue (10042727) and tongue pruritus (10070072) were more common among COVID-19 vaccinees and females as compared to seasonal influenza vaccinees and males. Interestingly, Maltezou et al. concluded that anaphylactic reactions rates are the lowest after COVID-19 vaccines (10.67 cases per one million doses) as compared with rabies, tick-borne encephalitis, measles-mumps-rubella-varicella, and human papillomavirus vaccines (70.77, 20, 19.8, and 13.65 cases per one million doses, respectively) (24). Earlier VAERS-based analyses indicated that anaphylactic rates were higher following BNT162b2 than mRNA-1273 vaccine, which is consistent with our findings (25, 26). Female predominance for post-vaccination allergic reactions was attributed to polyethene glycol hypersensitivity which was found to be higher among females than males (27, 28).

Other allergic AEs such as oral paraesthesia and oral hypoaesthesia were commonly reported among COVID-19 vaccinees and females. In the product assessment report of the mRNA-1273 vaccine published by the European Medicines Agency (EMA), oral paraesthesia was mentioned among the rare AEs (29). Likewise, mouth-tingling was enlisted as a rare allergic AE by regulators and professional societies in other countries (30).

Sporadic case reports/series for oral mucosa-related AEs following COVID-19 vaccination had been published during the previous months, calling for further investigation about the potential link between those AEs and the vaccine-elicited immune response (4). Oral lichen planus (OLP) was diagnosed in recently vaccinated individuals who received mRNA- (BNT162b2) and viral vector-based vaccines (Ad26.COV2.S) and whose medical anamneses and serological investigations ruled out suspected infections and allergens (31–33). Therefore, Hertel et al. performed a retrospective analysis for COVID-19 vaccinated *vs*. non-vaccinated cohorts of normal and overweight individuals and figured out that OLP incidence was higher in the vaccinated group (0.067 vs. 0.027%; *Sig*. < 0.001) (34). Contrarily, our current analysis found that OLP prevalence was lower among COVID-19 than among seasonal influenza vaccinees (0.006 vs. 0.014%; *Sig*. = 0.331). A recent meta-analysis of OLP point prevalence exhibited remarkably higher levels (0.89% for general populations and 0.98% for clinical patients) than reported after vaccination (35).

Oral herpes zoster (OHZ) is one of the suggested mucosa-related AEs empirically diagnosed in vaccinees with an irrelevant underlying anamnesis who received BNT162b2 (36). However, the pathophysiologic pathway remained unclear for how COVID-19 vaccines can trigger OHZ reactivation and the investigators warned the clinical and scientific communities from misdiagnosing OHZ as oral herpes (37). Prevalence of oral herpes (MedDRA ID: 10067152) that may include both herpes simplex and herpes zoster infections was significantly higher in the COVID-19 (0.189 vs. 0.086%; *Sig*. = 0.050) than seasonal influenza group. Within the COVID-19 group, prevalence of oral herpes was significantly higher among females (0.224 vs. 0.088%; *Sig*. < 0.001) and older age group (0.211 vs. 0.119%; *Sig*. < 0.001), and it was not significantly different between primer *vs*. booster doses (0.182 vs. 0.145%; *Sig*. = 0.061) or among vaccine brands. Female sex and older age predominance correspond with the current US demographics of oral herpes infection (38).

Aphthous stomatitis was suspected to be linked with recent COVID-19 vaccination in a few reported cases where patients presented with non-specific oral ulcers (39, 40). Unlike oral herpes, the prevalence of aphthous stomatitis was not significantly higher among COVID-19 vaccinees (0.168 vs. 0.158%; *Sig*. = 0.829); even though it resembled oral herpes in female and older age predominance and lack of preference for a particular dose or vaccine brand.

### Limitations

Several limitations should be taken into consideration while reading our analysis. Firstly, it is based on a passive surveillance database which is usually biased toward the less common moderate-to-severe AEs rather than the common mild AEs. Secondly, passive surveillance systems do not provide accurate epidemiologic estimates such as prevalence or incidence of the reported AEs; instead, they are used as an early alerting tool. Thirdly, most oral conditions, including neurological and mucosal AEs do not have background estimates that would have enabled us to perform the conventional observed-to-expected (O:E) analysis; therefore, we had to compare COVID-19 rates with another preemptively similar vaccine, i.e., seasonal influenza. Fourthly, the reported AEs were not systematically classified according to their exact onset, i.e., the interval between injection and AE emergence, by the VAERS database. Therefore, it is imperative that future research on oral AEs should record their onset to help determine the required resources for their management.

### Strengths

Heretofore, this analysis is the first to provide population-based evidence for COVID-19 AEs that might affect oral cavity structures and functions. It also suggests a *de novo* methodology that can be used for evaluating oral AEs of other vaccines e.g., H1N1, human papillomavirus and herpes zoster vaccines.

### Implications

By reading the findings of our analysis, dentists and dental team members can become more knowledgeable about the prevalence, severity, and prognosis of oral AEs that might arise after vaccination. Dentists are seen as trustworthy information sources by their patients and their knowledge and attitudes toward vaccine effectiveness and safety play a crucial role in enhancing the public uptake of vaccines. Moreover, oral health specialists should be actively engaged in the pharmacovigilance process as pragmatic point-of-care diagnostic schemes are urgently needed to improve the reporting quality of oral AEs—the Brighton Collaboration scheme for anaphylaxis diagnosis can be taken as an example. Finally, the general public should be reassured that the present analysis found that most COVID-19 oral AEs greatly resemble those of seasonal influenza vaccines and may not require particular attention except for those emerging immediately after the shot as they can be anaphylactic reactions.

## Conclusion

Within the limitations of this study, COVID-19 vaccines were found to be associated with rare oral AEs that were predominantly similar to those emerging following seasonal influenza vaccines. The most reported oral AEs were oral paresthesia (mouth-tingling), lip swelling, and ageusia, representing various pathophysiologic pathways that remain unclear. Taste-related AEs should be acknowledged in the context of the COVID-19 pandemic and the public should be adequately informed about a potential taste dysfunction after receiving the COVID-19 vaccination. Dentists and dental teams need to be aware of the prevalence, severity, and prognosis of oral AEs to inform their patients and increase public confidence in vaccines.

## Data Availability Statement

Publicly available datasets were analyzed in this study. This data can be found at: https://wonder.cdc.gov/vaers.html.

## Author Contributions

AR: conceptualization, methodology, formal analysis, and project administration. AR and SA: validation. AR, AP, and EK: writing—original draft preparation. SA: writing—review and editing and funding acquisition. All authors have read and agreed to the published version of the manuscript.

## Funding

The work of AR was supported by Masaryk University Grant Nos. MUNI/IGA/1104/2021 and MUNI/A/1402/2021.

## Conflict of Interest

The authors declare that the research was conducted in the absence of any commercial or financial relationships that could be construed as a potential conflict of interest.

## Publisher's Note

All claims expressed in this article are solely those of the authors and do not necessarily represent those of their affiliated organizations, or those of the publisher, the editors and the reviewers. Any product that may be evaluated in this article, or claim that may be made by its manufacturer, is not guaranteed or endorsed by the publisher.

## References

[1] Santos JA Normando AGC Silva RLC Acevedo AC Canto GDL Sugaya N. Oral manifestations in patients with COVID-19: a 6-month update. J Dent Res. (2021) 100:1321–9. 10.1177/00220345211029637 34324825

[2] Meng L Hua F Bian Z. Coronavirus disease 2019 (COVID-19): emerging and future challenges for dental and oral medicine. J Dent Res. (2020) 99:481–7. 10.1177/0022034520914246 32162995 PMC7140973

[3] Machingaidze S Wiysonge CS. Understanding COVID-19 vaccine hesitancy. Nat Med. (2021) 27:1338–9. 10.1038/s41591-021-01459-7 34272500

[4] Chun Y Jang J Jo JH Park JW. Various painful oral adverse reactions following COVID-19 vaccination: a case series. BMC Oral Health. (2022) 22:64. 10.1186/s12903-022-02100-w 35260129 PMC8902844

[5] CDC. CDC Wonder. Vaccine Advers Event Report System. CDC (2022). Available online at: https://wonder.cdc.gov/controller/datarequest/D8 (accessed May 25, 2022).

[6] ICH. MedDRA Hierarchy. Medical Dictionary Regulation Activites. ICH (2022). Available online at: https://www.meddra.org/how-to-use/basics/hierarchy (accessed May 25, 2022).

[7] CDC. Similarities and Differences between Flu and COVID-19. Influenza (2022). Available online at: https://www.cdc.gov/flu/symptoms/flu-vs-covid19.htm (accessed May 25, 2022).

[8] CIA. United States. World Factb. CIA (2022). Available online at: https://www.cia.gov/the-world-factbook/countries/united-states/ (accessed May 25, 2022).

[9] Cohen SR Gao DX Kahn JS Rosmarin D. Comparison of constitutional and dermatologic side effects between COVID-19 and non-COVID-19 vaccines: review of a publicly available database of vaccine side effects. J Am Acad Dermatol. (2022) 86:248–9. 10.1016/j.jaad.2021.09.044 34592382 PMC8482701

[10] Montano D. Frequency and associations of adverse reactions of COVID-19 Vaccines reported to pharmacovigilance systems in the European Union and the United States. Front Public Health. (2022) 9:2237. 10.3389/fpubh.2021.756633 35186864 PMC8850379

[11] Riad A Abdulqader H Morgado M Domnori S Koščík M Mendes JJ. Global prevalence and drivers of dental students' COVID-19 vaccine hesitancy. Vaccines. (2021) 9:566. 10.3390/vaccines9060566 34072500 PMC8226539

[12] CDC. Symptoms of COVID-19. CDC (2022). Available online at: https://www.cdc.gov/coronavirus/2019-ncov/symptoms-testing/symptoms.html (accessed May 10, 2022).

[13] Hannum ME Koch RJ Ramirez VA Marks SS Toskala AK Herriman RD. Taste loss as a distinct symptom of COVID-19: a systematic review and meta-analysis. Chem Senses. (2022) 47:bjac001. 10.1093/chemse/bjac001 35171979 PMC8849313

[14] Aziz M Goyal H Haghbin H Lee-Smith WM Gajendran M Perisetti A. The association of “loss of smell” to COVID-19: a systematic review and meta-analysis. Am J Med Sci. (2021) 361:216–25. 10.1016/j.amjms.2020.09.017 33349441 PMC7604015

[15] Alhaidari F Almuhaideb A Alsunaidi S Ibrahim N Aslam N Khan IU. E-Triage systems for COVID-19 outbreak: review and recommendations. Sensors. (2021) 21:2845. 10.3390/s21082845 33920744 PMC8072881

[16] Lechien JR Diallo AO Dachy B Le Bon SD Maniaci A Vaira LA. COVID-19: post-vaccine smell and taste disorders: report of 6 cases: *Ear Nose Throat J*. (2021) 1:01455613211033125. 10.1177/01455613211033125 34467793

[17] Alkhotani AM Alsindi TS Alqurashi AA Masarit RM Gazzaz RT Saggat RZ. Public awareness of the neurological manifestation of COVID-19 in Saudi Arabia. Neurosci J. (2022) 27:10–5. 10.17712/nsj.2022.1.20210089 35017285 PMC9037562

[18] Aldrees T Almatrafi S Aldriweesh T Mokhatrish M Salamh A Alkholaiwi F. Medical students' awareness of smell loss as a predictor for Coronavirus disease 2019. Front Public Health. (2020) 8:863. 10.3389/fpubh.2020.597897 33363090 PMC7756011

[19] Riad A Pokorná A Attia S Klugarová J Koščík M Klugar M. Prevalence of COVID-19 vaccine side effects among healthcare workers in the Czech Republic. J Clin Med. (2021) 10:1428. 10.3390/jcm10071428 33916020 PMC8037149

[20] Mazur M Duś-Ilnicka I Jedliński M Ndokaj A Janiszewska-Olszowska J Ardan R. Facial and oral manifestations following COVID-19 vaccination: a survey-based study and a first perspective. Int J Environ Res Public Health. (2021) 18:4965. 10.3390/ijerph18094965 34066995 PMC8125066

[21] Riad A Hocková B Kantorová L Slávik R Spurná L Stebel A. Side effects of mRNA-based COVID-19 vaccine: nationwide phase IV study among healthcare workers in Slovakia. Pharm. (2021) 14:873. 10.3390/ph14090873 34577573 PMC8466035

[22] Lapić I Šegulja D Rogić D. Assessment of salivary antibody response to BNT162b2 mRNA COVID-19 vaccination. J Med Virol. (2021) 93:5257–9. 10.1002/jmv.27096 34009653 PMC8242469

[23] Azzi L Dalla Gasperina D Veronesi G Shallak M Ietto G Iovino D. Mucosal immune response in BNT162b2 COVID-19 vaccine recipients. eBioMedicine. (2022) 75:103788. 10.1016/j.ebiom.2021.103788 34954658 PMC8718969

[24] Maltezou HC Anastassopoulou C Hatziantoniou S Poland GA Tsakris A. Anaphylaxis rates associated with COVID-19 vaccines are comparable to those of other vaccines. Vaccine. (2022) 40:183–6. 10.1016/j.vaccine.2021.11.066 34863620 PMC8626274

[25] Shimabukuro T Nair N. Allergic reactions including anaphylaxis after receipt of the first dose of Pfizer-BioNTech COVID-19 vaccine. JAMA. (2021) 325:780–1. 10.1001/jama.2021.0600 33475702 PMC8892260

[26] CDC. Allergic reactions including anaphylaxis after receipt of the first dose of moderna COVID-19 vaccine — United States, December 21, 2020–January 10, 2021. Morb Mortal Wkly Rep. (2021) 21:1326–31. 10.1111/ajt.16517 33507892 PMC7842812

[27] Somiya M Mine S Yasukawa K Ikeda S. Sex differences in the incidence of anaphylaxis to LNP-mRNA COVID-19 vaccines. Vaccine. (2021) 39:3313. 10.1016/j.vaccine.2021.04.066 34020815 PMC8101867

[28] Yang Q Jacobs TM McCallen JD Moore DT Huckaby JT Edelstein JN. Analysis of pre-existing IgG and IgM antibodies against polyethylene glycol (PEG) in the general population. Anal Chem. (2016) 88:11804–12. 10.1021/acs.analchem.6b03437 27804292 PMC6512330

[29] EMA. Assessment Report: COVID-19 Vaccine Moderna. Union Register Medicinal Products (2021). Available online at: https://ec.europa.eu/health/documents/community-register/html/h1507.htm (accessed May 25, 2022).

[30] ASCIA. Allergy, Immunodeficiency, Autoimmunity and COVID-19 Vaccination Frequently Asked Questions (FAQ). COVID-19 Vaccin FAQ (2022). Available online at: https://www.allergy.org.au/patients/ascia-covid-19-vaccination-faq (accessed May 25, 2022).

[31] Troeltzsch M Gogl M Berndt R Troeltzsch M. Oral lichen planus following the administration of vector-based COVID-19 vaccine (Ad26.COV2.S). Oral Dis. (2021). 10.1111/odi.14025 34543493 PMC8661663

[32] Kaomongkolgit R Sawangarun W. Oral lichen planus following mRNA COVID-19 vaccination. Oral Dis. (2022). 10.1111/odi.14182 35263820 PMC9115415

[33] Raccampo L Sembronio S Tel A Veronica C Robiony M. Oral lichen planus arising after BNT162b2 mRNA COVID-19 vaccine: report of two cases. Oral Surg Oral Med Oral Pathol Oral Radiol. (2022). 10.1016/j.oooo.2022.04.003 PMC8995197 35851250

[34] Hertel M Schmidt-Westhausen A-M Wendy S Heiland M Nahles S Preissner R. Onset of oral lichenoid lesions and oral lichen planus following COVID-19 vaccination: a retrospective analysis of about 300,000 vaccinated patients. Vaccines. (2022) 10:480. 10.3390/vaccines10030480 35335112 PMC8951494

[35] Li C Tang X Zheng X Ge S Wen H Lin X. Global prevalence and incidence estimates of oral lichen planus: a systematic review and meta-analysis. JAMA Dermatology. (2020) 156:172. 10.1001/jamadermatol.2019.3797 31895418 PMC6990670

[36] Fukuoka H Fukuoka N Kibe T Tubbs RS Iwanaga J. Oral herpes zoster infection following COVID-19 vaccination: a report of five cases. Cureus. (2021) 13:e19433. 10.7759/cureus.19433 34909338 PMC8663753

[37] Iwanaga J Fukuoka H Fukuoka N Yutori H Ibaragi S Tubbs RS. A narrative review and clinical anatomy of herpes zoster infection following COVID-19 vaccination. Clin Anat. (2022) 35:45–51. 10.1002/ca.23790 34554601 PMC8652627

[38] McQuillan G Kruszon-Moran D Flagg EW Paulose-Ram R. Prevalence of Herpes Simplex Virus Type 1 Type 2 in Persons Aged 14–49: United States, 2015–2016. National Center Health Statistics Data Briefs (2018). Available online at: https://www.cdc.gov/nchs/products/databriefs/db304.htm (accessed May 10, 2022).

[39] Maeda K Yamashita D Takenobu T. Ulcers on the bilateral palate mucosa following mRNA-based vaccination for coronavirus disease 2019 (COVID-19): a case report. J Stomatol Oral Maxillofac Surg. (2022) 123:283–6. 10.1016/j.jormas.2022.01.013 35114426 PMC8805911

[40] Kim HK Kim ME. A case of aphthous stomatitis in a healthy adult following COVID-19 vaccination: clinical reasoning. J Oral Med Pain. (2022) 47:62–6. 10.14476/jomp.2022.47.1.62

