# Supplementary material for: Oral Adverse Events Following COVID-19 Vaccination: Analysis of VAERS Reports
Source: Front Public Health. 2022 Jul 11;10:952781. doi: 10.3389/fpubh.2022.952781 (PMC9309565; doi:10.3389/fpubh.2022.952781)
Supplement: Supplementary file 1 [file Table_1.DOCX]

**Table S1.** Initial List of Oral Adverse Events Following Immunization (AEFI) Based on Our Proposed Anatomo-physiological Scheme (*n* = 310)

| 1 | Teeth OR Tooth OR Dentition | >10006514 (BRUXISM) |
| --- | --- | --- |
| 2 | Teeth OR Tooth OR Dentition | >10012318 (DENTAL CARIES) |
| 3 | Teeth OR Tooth OR Dentition | >10054217 (DENTAL DISCOMFORT) |
| 4 | Teeth OR Tooth OR Dentition | >10078276 (DENTAL PARAESTHESIA) |
| 5 | Teeth OR Tooth OR Dentition | >10016018 (FACE AND MOUTH X-RAY) |
| 6 | Teeth OR Tooth OR Dentition | >10082426 (HYPERAESTHESIA TEETH) |
| 7 | Teeth OR Tooth OR Dentition | >10051780 (HYPOAESTHESIA TEETH) |
| 8 | Teeth OR Tooth OR Dentition | >10065612 (LOOSE TOOTH) |
| 9 | Teeth OR Tooth OR Dentition | >10061274 (MALOCCLUSION) |
| 10 | Teeth OR Tooth OR Dentition | >10076265 (MALPOSITIONED TEETH) |
| 11 | Teeth OR Tooth OR Dentition | >10037464 (PULPITIS DENTAL) |
| 12 | Teeth OR Tooth OR Dentition | >10040012 (SENSITIVITY OF TEETH) |
| 13 | Teeth OR Tooth OR Dentition | >10043173 (TEETH BRITTLE) |
| 14 | Teeth OR Tooth OR Dentition | >10043183 (TEETHING) |
| 15 | Teeth OR Tooth OR Dentition | >10044016 (TOOTH ABSCESS) |
| 16 | Teeth OR Tooth OR Dentition | >10065953 (TOOTH AVULSION) |
| 17 | Teeth OR Tooth OR Dentition | >10072665 (TOOTH DEMINERALISATION) |
| 18 | Teeth OR Tooth OR Dentition | >10044029 (TOOTH DEPOSIT) |
| 19 | Teeth OR Tooth OR Dentition | >10044030 (TOOTH DEVELOPMENT DISORDER) |
| 20 | Teeth OR Tooth OR Dentition | >10044032 (TOOTH DISCOLOURATION) |
| 21 | Teeth OR Tooth OR Dentition | >10079745 (TOOTH DISLOCATION) |
| 22 | Teeth OR Tooth OR Dentition | >10044034 (TOOTH DISORDER) |
| 23 | Teeth OR Tooth OR Dentition | >10044038 (TOOTH EROSION) |
| 24 | Teeth OR Tooth OR Dentition | >10062132 (TOOTH EXTRACTION) |
| 25 | Teeth OR Tooth OR Dentition | >10062544 (TOOTH FRACTURE) |
| 26 | Teeth OR Tooth OR Dentition | >10044041 (TOOTH HYPOPLASIA) |
| 27 | Teeth OR Tooth OR Dentition | >10044042 (TOOTH IMPACTED) |
| 28 | Teeth OR Tooth OR Dentition | >10048762 (TOOTH INFECTION) |
| 29 | Teeth OR Tooth OR Dentition | >10044043 (TOOTH INJURY) |
| 30 | Teeth OR Tooth OR Dentition | >10044044 (TOOTH LOSS) |
| 31 | Teeth OR Tooth OR Dentition | >10044046 (TOOTH MALFORMATION) |
| 32 | Teeth OR Tooth OR Dentition | >10044051 (TOOTH REPAIR) |
| 33 | Teeth OR Tooth OR Dentition | >10044052 (TOOTH RESORPTION) |
| 34 | Teeth OR Tooth OR Dentition | >10085637 (TOOTH RESTORATION) |
| 35 | Teeth OR Tooth OR Dentition | >10064946 (TOOTH SOCKET HAEMORRHAGE) |
| 36 | Teeth OR Tooth OR Dentition | >10044055 (TOOTHACHE) |
| 37 | Teeth OR Tooth OR Dentition | >10071180 (TRAUMATIC TOOTH DISPLACEMENT) |
| 38 | Teeth OR Tooth OR Dentition | >10047991 (WISDOM TEETH REMOVAL) |
| 39 | Teeth OR Tooth OR Dentition | >10052955 (X-RAY DENTAL) |
| 40 | Teeth OR Tooth OR Dentition | >10079993 (X-RAY DENTAL ABNORMAL) |
| 41 | Teeth OR Tooth OR Dentition | >10079994 (X-RAY DENTAL NORMAL) |
| 42 | Lips OR Lip OR Labial | >10082548 (ANAESTHESIA ORAL) |
| 43 | Lips OR Lip OR Labial | >10002958 (APHTHOUS STOMATITIS) |
| 44 | Lips OR Lip OR Labial | >10002959 (APHTHOUS ULCER) |
| 45 | Lips OR Lip OR Labial | >10004788 (BIOPSY LIP) |
| 46 | Lips OR Lip OR Labial | >10004789 (BIOPSY LIP ABNORMAL) |
| 47 | Lips OR Lip OR Labial | >10006784 (BURNING SENSATION) |
| 48 | Lips OR Lip OR Labial | >10049047 (CHAPPED LIPS) |
| 49 | Lips OR Lip OR Labial | >10008417 (CHEILITIS) |
| 50 | Lips OR Lip OR Labial | >10070782 (CHEMICAL BURN OF ORAL CAVITY) |
| 51 | Lips OR Lip OR Labial | >10009259 (CLEFT LIP) |
| 52 | Lips OR Lip OR Labial | >10009260 (CLEFT LIP AND PALATE) |
| 53 | Lips OR Lip OR Labial | >10056999 (CLEFT LIP REPAIR) |
| 54 | Lips OR Lip OR Labial | >10066241 (COMPULSIVE LIP BITING) |
| 55 | Lips OR Lip OR Labial | >10057371 (HYPOAESTHESIA ORAL) |
| 56 | Lips OR Lip OR Labial | >10076772 (LABIAL TIE) |
| 57 | Lips OR Lip OR Labial | >10061523 (LIP AND/OR ORAL CAVITY CANCER) |
| 58 | Lips OR Lip OR Labial | >10024535 (LIP AND/OR ORAL CAVITY CANCER RECURRENT) |
| 59 | Lips OR Lip OR Labial | >10049307 (LIP BLISTER) |
| 60 | Lips OR Lip OR Labial | >10079830 (LIP COSMETIC PROCEDURE) |
| 61 | Lips OR Lip OR Labial | >10024549 (LIP DISCOLOURATION) |
| 62 | Lips OR Lip OR Labial | >10048470 (LIP DISORDER) |
| 63 | Lips OR Lip OR Labial | >10024552 (LIP DRY) |
| 64 | Lips OR Lip OR Labial | >10051992 (LIP EROSION) |
| 65 | Lips OR Lip OR Labial | >10080124 (LIP ERYTHEMA) |
| 66 | Lips OR Lip OR Labial | >10064482 (LIP EXFOLIATION) |
| 67 | Lips OR Lip OR Labial | >10066304 (LIP HAEMATOMA) |
| 68 | Lips OR Lip OR Labial | >10049297 (LIP HAEMORRHAGE) |
| 69 | Lips OR Lip OR Labial | >10065755 (LIP INFECTION) |
| 70 | Lips OR Lip OR Labial | >10055082 (LIP INJURY) |
| 71 | Lips OR Lip OR Labial | >10024555 (LIP LESION EXCISION) |
| 72 | Lips OR Lip OR Labial | >10062038 (LIP NEOPLASM) |
| 73 | Lips OR Lip OR Labial | >10024558 (LIP OEDEMA) |
| 74 | Lips OR Lip OR Labial | >10052977 (LIP OPERATION) |
| 75 | Lips OR Lip OR Labial | >10024561 (LIP PAIN) |
| 76 | Lips OR Lip OR Labial | >10070721 (LIP PRURITUS) |
| 77 | Lips OR Lip OR Labial | >10024565 (LIP REPAIR) |
| 78 | Lips OR Lip OR Labial | >10082767 (LIP SCAB) |
| 79 | Lips OR Lip OR Labial | >10024570 (LIP SWELLING) |
| 80 | Lips OR Lip OR Labial | >10024572 (LIP ULCERATION) |
| 81 | Lips OR Lip OR Labial | >10049294 (MOUTH INJURY) |
| 82 | Lips OR Lip OR Labial | >10078170 (ORAL CONTUSION) |
| 83 | Lips OR Lip OR Labial | >10030973 (ORAL DISCOMFORT) |
| 84 | Lips OR Lip OR Labial | >10050820 (ORAL DYSAESTHESIA) |
| 85 | Lips OR Lip OR Labial | >10067152 (ORAL HERPES) |
| 86 | Lips OR Lip OR Labial | >10084426 (ORAL MUCOSAL SCAR) |
| 87 | Lips OR Lip OR Labial | >10056674 (ORAL PUSTULE) |
| 88 | Lips OR Lip OR Labial | >10057372 (PARAESTHESIA ORAL) |
| 89 | Mandible OR Mandibular | >10015690 (EXOSTOSIS OF JAW) |
| 90 | Mandible OR Mandibular | >10016018 (FACE AND MOUTH X-RAY) |
| 91 | Mandible OR Mandibular | >10016019 (FACE AND MOUTH X-RAY ABNORMAL) |
| 92 | Mandible OR Mandibular | >10016020 (FACE AND MOUTH X-RAY NORMAL) |
| 93 | Mandible OR Mandibular | >10023149 (JAW FRACTURE) |
| 94 | Mandible OR Mandibular | >10059242 (JAW OPERATION) |
| 95 | Mandible OR Mandibular | >10025188 (LYMPHADENITIS) |
| 96 | Mandible OR Mandibular | >10025197 (LYMPHADENOPATHY) |
| 97 | Mandible OR Mandibular | >10079878 (MANDIBULAR MASS) |
| 98 | Mandible OR Mandibular | >10027541 (MICROGENIA) |
| 99 | Mandible OR Mandibular | >10067954 (OROMANDIBULAR DYSTONIA) |
| 100 | Mandible OR Mandibular | >10039394 (SALIVARY GLAND CALCULUS) |
| 101 | Mandible OR Mandibular | >10061934 (SALIVARY GLAND CANCER) |
| 102 | Mandible OR Mandibular | >10057002 (SALIVARY GLAND MASS) |
| 103 | Mandible OR Mandibular | >10040628 (SIALOADENITIS) |
| 104 | Mandible OR Mandibular | >10050123 (TEMPOROMANDIBULAR JOINT SURGERY) |
| 105 | Mandible OR Mandibular | >10043220 (TEMPOROMANDIBULAR JOINT SYNDROME) |
| 106 | Saliva OR Salivary Gland | >10000305 (ABSCESS OF SALIVARY GLAND) |
| 107 | Saliva OR Salivary Gland | >10073369 (ACINIC CELL CARCINOMA OF SALIVARY GLAND) |
| 108 | Saliva OR Salivary Gland | >10002016 (AMYLASE INCREASED) |
| 109 | Saliva OR Salivary Gland | >10003068 (APTYALISM) |
| 110 | Saliva OR Salivary Gland | >10004453 (BENIGN SALIVARY GLAND NEOPLASM) |
| 111 | Saliva OR Salivary Gland | >10004865 (BIOPSY SALIVARY GLAND) |
| 112 | Saliva OR Salivary Gland | >10004866 (BIOPSY SALIVARY GLAND ABNORMAL) |
| 113 | Saliva OR Salivary Gland | >10004867 (BIOPSY SALIVARY GLAND NORMAL) |
| 114 | Saliva OR Salivary Gland | >10075243 (NONINFECTIVE SIALOADENITIS) |
| 115 | Saliva OR Salivary Gland | >10039379 (SALIVA ALTERED) |
| 116 | Saliva OR Salivary Gland | >10049069 (SALIVA DISCOLOURATION) |
| 117 | Saliva OR Salivary Gland | >10056681 (SALIVARY DUCT INFLAMMATION) |
| 118 | Saliva OR Salivary Gland | >10039386 (SALIVARY DUCT OBSTRUCTION) |
| 119 | Saliva OR Salivary Gland | >10039388 (SALIVARY DUCT STENOSIS) |
| 120 | Saliva OR Salivary Gland | >10051636 (SALIVARY GLAND ADENOMA) |
| 121 | Saliva OR Salivary Gland | >10039394 (SALIVARY GLAND CALCULUS) |
| 122 | Saliva OR Salivary Gland | >10061934 (SALIVARY GLAND CANCER) |
| 123 | Saliva OR Salivary Gland | >10052248 (SALIVARY GLAND CYST) |
| 124 | Saliva OR Salivary Gland | >10061935 (SALIVARY GLAND DISORDER) |
| 125 | Saliva OR Salivary Gland | >10039408 (SALIVARY GLAND ENLARGEMENT) |
| 126 | Saliva OR Salivary Gland | >10071363 (SALIVARY GLAND INDURATION) |
| 127 | Saliva OR Salivary Gland | >10057002 (SALIVARY GLAND MASS) |
| 128 | Saliva OR Salivary Gland | >10060870 (SALIVARY GLAND MUCOCOELE) |
| 129 | Saliva OR Salivary Gland | >10061497 (SALIVARY GLAND NEOPLASM) |
| 130 | Saliva OR Salivary Gland | >10064759 (SALIVARY GLAND OPERATION) |
| 131 | Saliva OR Salivary Gland | >10039421 (SALIVARY GLAND PAIN) |
| 132 | Saliva OR Salivary Gland | >10039424 (SALIVARY HYPERSECRETION) |
| 133 | Saliva OR Salivary Gland | >10039426 (SALIVARY SCAN) |
| 134 | Saliva OR Salivary Gland | >10040628 (SIALOADENITIS) |
| 135 | Palate OR Palatal | >10004833 (BIOPSY PALATE) |
| 136 | Palate OR Palatal | >10009260 (CLEFT LIP AND PALATE) |
| 137 | Palate OR Palatal | >10056999 (CLEFT LIP REPAIR) |
| 138 | Palate OR Palatal | >10009269 (CLEFT PALATE) |
| 139 | Palate OR Palatal | >10067418 (ORAL MUCOSAL ERYTHEMA) |
| 140 | Palate OR Palatal | >10031009 (ORAL PAIN) |
| 141 | Palate OR Palatal | >10052453 (PALATAL DISORDER) |
| 142 | Palate OR Palatal | >10056998 (PALATAL OEDEMA) |
| 143 | Palate OR Palatal | >10072012 (PALATAL PALSY) |
| 144 | Palate OR Palatal | >10074403 (PALATAL SWELLING) |
| 145 | Palate OR Palatal | >10077519 (PALATAL ULCER) |
| 146 | Palate OR Palatal | >10079812 (PALATE INJURY) |
| 147 | Tongue OR Lingual OR Glossia | >10058835 (ACQUIRED MACROGLOSSIA) |
| 148 | Tongue OR Lingual OR Glossia | >10082548 (ANAESTHESIA ORAL) |
| 149 | Tongue OR Lingual OR Glossia | >10049243 (ANKYLOGLOSSIA ACQUIRED) |
| 150 | Tongue OR Lingual OR Glossia | >10049244 (ANKYLOGLOSSIA CONGENITAL) |
| 151 | Tongue OR Lingual OR Glossia | >10069085 (ATROPHIC GLOSSITIS) |
| 152 | Tongue OR Lingual OR Glossia | >10003712 (ATROPHY OF TONGUE PAPILLAE) |
| 153 | Tongue OR Lingual OR Glossia | >10004891 (BIOPSY TONGUE) |
| 154 | Tongue OR Lingual OR Glossia | >10004892 (BIOPSY TONGUE ABNORMAL) |
| 155 | Tongue OR Lingual OR Glossia | >10075532 (BURN ORAL CAVITY) |
| 156 | Tongue OR Lingual OR Glossia | >10061077 (CONGENITAL TONGUE ANOMALY) |
| 157 | Tongue OR Lingual OR Glossia | >10018386 (GLOSSITIS) |
| 158 | Tongue OR Lingual OR Glossia | >10018388 (GLOSSODYNIA) |
| 159 | Tongue OR Lingual OR Glossia | >10020893 (HYPERTROPHY OF TONGUE PAPILLAE) |
| 160 | Tongue OR Lingual OR Glossia | >10057371 (HYPOAESTHESIA ORAL) |
| 161 | Tongue OR Lingual OR Glossia | >10070963 (INFECTIVE GLOSSITIS) |
| 162 | Tongue OR Lingual OR Glossia | >10024396 (LEUKOPLAKIA ORAL) |
| 163 | Tongue OR Lingual OR Glossia | >10025391 (MACROGLOSSIA) |
| 164 | Tongue OR Lingual OR Glossia | >10078170 (ORAL CONTUSION) |
| 165 | Tongue OR Lingual OR Glossia | >10050820 (ORAL DYSAESTHESIA) |
| 166 | Tongue OR Lingual OR Glossia | >10077632 (ORAL HAEMANGIOMA) |
| 167 | Tongue OR Lingual OR Glossia | >10067954 (OROMANDIBULAR DYSTONIA) |
| 168 | Tongue OR Lingual OR Glossia | >10057372 (PARAESTHESIA ORAL) |
| 169 | Tongue OR Lingual OR Glossia | >10035630 (PLICATED TONGUE) |
| 170 | Tongue OR Lingual OR Glossia | >10037076 (PROTRUSION TONGUE) |
| 171 | Tongue OR Lingual OR Glossia | >10074686 (SCALLOPED TONGUE) |
| 172 | Tongue OR Lingual OR Glossia | >10041865 (SQUAMOUS CELL CARCINOMA OF THE TONGUE) |
| 173 | Tongue OR Lingual OR Glossia | >10081491 (STIFF TONGUE) |
| 174 | Tongue OR Lingual OR Glossia | >10042128 (STOMATITIS) |
| 175 | Tongue OR Lingual OR Glossia | >10051495 (STRAWBERRY TONGUE) |
| 176 | Tongue OR Lingual OR Glossia | >10042727 (SWOLLEN TONGUE) |
| 177 | Tongue OR Lingual OR Glossia | >10066991 (TONGUE ABSCESS) |
| 178 | Tongue OR Lingual OR Glossia | >10055028 (TONGUE ATROPHY) |
| 179 | Tongue OR Lingual OR Glossia | >10050467 (TONGUE BITING) |
| 180 | Tongue OR Lingual OR Glossia | >10043941 (TONGUE BLACK HAIRY) |
| 181 | Tongue OR Lingual OR Glossia | >10043942 (TONGUE BLISTERING) |
| 182 | Tongue OR Lingual OR Glossia | >10055109 (TONGUE CANCER METASTATIC) |
| 183 | Tongue OR Lingual OR Glossia | >10071251 (TONGUE CANCER RECURRENT) |
| 184 | Tongue OR Lingual OR Glossia | >10043945 (TONGUE COATED) |
| 185 | Tongue OR Lingual OR Glossia | >10051879 (TONGUE CYST) |
| 186 | Tongue OR Lingual OR Glossia | >10043949 (TONGUE DISCOLOURATION) |
| 187 | Tongue OR Lingual OR Glossia | >10077855 (TONGUE DISCOMFORT) |
| 188 | Tongue OR Lingual OR Glossia | >10043951 (TONGUE DISORDER) |
| 189 | Tongue OR Lingual OR Glossia | >10049713 (TONGUE DRY) |
| 190 | Tongue OR Lingual OR Glossia | >10052002 (TONGUE ERUPTION) |
| 191 | Tongue OR Lingual OR Glossia | >10079075 (TONGUE ERYTHEMA) |
| 192 | Tongue OR Lingual OR Glossia | >10064488 (TONGUE EXFOLIATION) |
| 193 | Tongue OR Lingual OR Glossia | >10075845 (TONGUE FUNGAL INFECTION) |
| 194 | Tongue OR Lingual OR Glossia | >10043957 (TONGUE GEOGRAPHIC) |
| 195 | Tongue OR Lingual OR Glossia | >10043959 (TONGUE HAEMATOMA) |
| 196 | Tongue OR Lingual OR Glossia | >10049870 (TONGUE HAEMORRHAGE) |
| 197 | Tongue OR Lingual OR Glossia | >10084548 (TONGUE INDURATION) |
| 198 | Tongue OR Lingual OR Glossia | >10059924 (TONGUE INJURY) |
| 199 | Tongue OR Lingual OR Glossia | >10043963 (TONGUE MOVEMENT DISTURBANCE) |
| 200 | Tongue OR Lingual OR Glossia | >10062129 (TONGUE NEOPLASM) |
| 201 | Tongue OR Lingual OR Glossia | >10043966 (TONGUE NEOPLASM MALIGNANT STAGE UNSPECIFIED) |
| 202 | Tongue OR Lingual OR Glossia | >10043967 (TONGUE OEDEMA) |
| 203 | Tongue OR Lingual OR Glossia | >10062130 (TONGUE OPERATION) |
| 204 | Tongue OR Lingual OR Glossia | >10043972 (TONGUE PARALYSIS) |
| 205 | Tongue OR Lingual OR Glossia | >10069164 (TONGUE PIGMENTATION) |
| 206 | Tongue OR Lingual OR Glossia | >10074778 (TONGUE POLYP) |
| 207 | Tongue OR Lingual OR Glossia | >10070072 (TONGUE PRURITUS) |
| 208 | Tongue OR Lingual OR Glossia | >10043977 (TONGUE ROUGH) |
| 209 | Tongue OR Lingual OR Glossia | >10043981 (TONGUE SPASM) |
| 210 | Tongue OR Lingual OR Glossia | >10082545 (TONGUE THRUST) |
| 211 | Tongue OR Lingual OR Glossia | >10043988 (TONGUE TIE OPERATION) |
| 212 | Tongue OR Lingual OR Glossia | >10043991 (TONGUE ULCERATION) |
| 213 | Tongue OR Lingual OR Glossia | >10079293 (TRANSIENT LINGUAL PAPILLITIS) |
| 214 | Tongue OR Lingual OR Glossia | >10080276 (TRICHOGLOSSIA) |
| 215 | Oral OR Mouth | >10000311 (ABSCESS ORAL) |
| 216 | Oral OR Mouth | >10082548 (ANAESTHESIA ORAL) |
| 217 | Oral OR Mouth | >10002509 (ANGULAR CHEILITIS) |
| 218 | Oral OR Mouth | >10002958 (APHTHOUS STOMATITIS) |
| 219 | Oral OR Mouth | >10002959 (APHTHOUS ULCER) |
| 220 | Oral OR Mouth | >10048479 (BUCCAL MUCOSAL ROUGHENING) |
| 221 | Oral OR Mouth | >10075532 (BURN ORAL CAVITY) |
| 222 | Oral OR Mouth | >10068065 (BURNING MOUTH SYNDROME) |
| 223 | Oral OR Mouth | >10008417 (CHEILITIS) |
| 224 | Oral OR Mouth | >10052250 (CIRCUMORAL OEDEMA) |
| 225 | Oral OR Mouth | >10081703 (CIRCUMORAL SWELLING) |
| 226 | Oral OR Mouth | >10075366 (COATING IN MOUTH) |
| 227 | Oral OR Mouth | >10061070 (CONGENITAL ORAL MALFORMATION) |
| 228 | Oral OR Mouth | >10013781 (DRY MOUTH) |
| 229 | Oral OR Mouth | >10016018 (FACE AND MOUTH X-RAY) |
| 230 | Oral OR Mouth | >10016019 (FACE AND MOUTH X-RAY ABNORMAL) |
| 231 | Oral OR Mouth | >10016020 (FACE AND MOUTH X-RAY NORMAL) |
| 232 | Oral OR Mouth | >10019113 (HAND-FOOT-AND-MOUTH DISEASE) |
| 233 | Oral OR Mouth | >10057371 (HYPOAESTHESIA ORAL) |
| 234 | Oral OR Mouth | >10063743 (HYPOPHAGIA) |
| 235 | Oral OR Mouth | >10024396 (LEUKOPLAKIA ORAL) |
| 236 | Oral OR Mouth | >10061523 (LIP AND/OR ORAL CAVITY CANCER) |
| 237 | Oral OR Mouth | >10024535 (LIP AND/OR ORAL CAVITY CANCER RECURRENT) |
| 238 | Oral OR Mouth | >10028017 (MOUTH BREATHING) |
| 239 | Oral OR Mouth | >10028020 (MOUTH CYST) |
| 240 | Oral OR Mouth | >10028024 (MOUTH HAEMORRHAGE) |
| 241 | Oral OR Mouth | >10049294 (MOUTH INJURY) |
| 242 | Oral OR Mouth | >10028032 (MOUTH PLAQUE) |
| 243 | Oral OR Mouth | >10075203 (MOUTH SWELLING) |
| 244 | Oral OR Mouth | >10028034 (MOUTH ULCERATION) |
| 245 | Oral OR Mouth | >10055670 (NECROTISING ULCERATIVE GINGIVOSTOMATITIS) |
| 246 | Oral OR Mouth | >10080668 (NOTHING BY MOUTH ORDER) |
| 247 | Oral OR Mouth | >10030110 (OEDEMA MOUTH) |
| 248 | Oral OR Mouth | >10068355 (ORAL ALLERGY SYNDROME) |
| 249 | Oral OR Mouth | >10065233 (ORAL BACTERIAL INFECTION) |
| 250 | Oral OR Mouth | >10076590 (ORAL BLOOD BLISTER) |
| 251 | Oral OR Mouth | >10030963 (ORAL CANDIDIASIS) |
| 252 | Oral OR Mouth | >10086211 (ORAL CAVITY EXAMINATION) |
| 253 | Oral OR Mouth | >10065720 (ORAL CAVITY FISTULA) |
| 254 | Oral OR Mouth | >10030970 (ORAL CONTRACEPTION) |
| 255 | Oral OR Mouth | >10078170 (ORAL CONTUSION) |
| 256 | Oral OR Mouth | >10030972 (ORAL DISCHARGE) |
| 257 | Oral OR Mouth | >10030973 (ORAL DISCOMFORT) |
| 258 | Oral OR Mouth | >10067621 (ORAL DISORDER) |
| 259 | Oral OR Mouth | >10050820 (ORAL DYSAESTHESIA) |
| 260 | Oral OR Mouth | >10079559 (ORAL DYSPLASIA) |
| 261 | Oral OR Mouth | >10061324 (ORAL FUNGAL INFECTION) |
| 262 | Oral OR Mouth | >10077632 (ORAL HAEMANGIOMA) |
| 263 | Oral OR Mouth | >10067152 (ORAL HERPES) |
| 264 | Oral OR Mouth | >10048685 (ORAL INFECTION) |
| 265 | Oral OR Mouth | >10063889 (ORAL INTAKE REDUCED) |
| 266 | Oral OR Mouth | >10030983 (ORAL LICHEN PLANUS) |
| 267 | Oral OR Mouth | >10083833 (ORAL LICHENOID REACTION) |
| 268 | Oral OR Mouth | >10064594 (ORAL MUCOSA EROSION) |
| 269 | Oral OR Mouth | >10074779 (ORAL MUCOSA HAEMATOMA) |
| 270 | Oral OR Mouth | >10030995 (ORAL MUCOSAL BLISTERING) |
| 271 | Oral OR Mouth | >10030996 (ORAL MUCOSAL DISCOLOURATION) |
| 272 | Oral OR Mouth | >10030997 (ORAL MUCOSAL ERUPTION) |
| 273 | Oral OR Mouth | >10067418 (ORAL MUCOSAL ERYTHEMA) |
| 274 | Oral OR Mouth | >10064487 (ORAL MUCOSAL EXFOLIATION) |
| 275 | Oral OR Mouth | >10062956 (ORAL MUCOSAL HYPERTROPHY) |
| 276 | Oral OR Mouth | >10030998 (ORAL MUCOSAL PETECHIAE) |
| 277 | Oral OR Mouth | >10084009 (ORAL MUCOSAL ROUGHENING) |
| 278 | Oral OR Mouth | >10082769 (ORAL MUCOSAL SCAB) |
| 279 | Oral OR Mouth | >10084426 (ORAL MUCOSAL SCAR) |
| 280 | Oral OR Mouth | >10061325 (ORAL NEOPLASM) |
| 281 | Oral OR Mouth | >10031009 (ORAL PAIN) |
| 282 | Oral OR Mouth | >10068322 (ORAL PAPILLOMA) |
| 283 | Oral OR Mouth | >10031010 (ORAL PAPULE) |
| 284 | Oral OR Mouth | >10077552 (ORAL PIGMENTATION) |
| 285 | Oral OR Mouth | >10052894 (ORAL PRURITUS) |
| 286 | Oral OR Mouth | >10083533 (ORAL PURPURA) |
| 287 | Oral OR Mouth | >10056674 (ORAL PUSTULE) |
| 288 | Oral OR Mouth | >10078906 (ORAL SOFT TISSUE BIOPSY) |
| 289 | Oral OR Mouth | >10061326 (ORAL SOFT TISSUE DISORDER) |
| 290 | Oral OR Mouth | >10051059 (ORAL SURGERY) |
| 291 | Oral OR Mouth | >10065234 (ORAL VIRAL INFECTION) |
| 292 | Oral OR Mouth | >10067950 (OROPHARYNGEAL BLISTERING) |
| 293 | Oral OR Mouth | >10067721 (OROPHARYNGEAL PLAQUE) |
| 294 | Oral OR Mouth | >10074403 (PALATAL SWELLING) |
| 295 | Oral OR Mouth | >10057372 (PARAESTHESIA ORAL) |
| 296 | Oral OR Mouth | >10034541 (PERIORAL DERMATITIS) |
| 297 | Oral OR Mouth | >10060870 (SALIVARY GLAND MUCOCOELE) |
| 298 | Oral OR Mouth | >10041857 (SQUAMOUS CELL CARCINOMA OF THE ORAL CAVITY) |
| 299 | Oral OR Mouth | >10042128 (STOMATITIS) |
| 300 | Oral OR Mouth | >10042135 (STOMATITIS NECROTISING) |
| 301 | Oral OR Mouth | >10052955 (X-RAY DENTAL) |
| 302 | Oral OR Mouth | >10079993 (X-RAY DENTAL ABNORMAL) |
| 303 | Oral OR Mouth | >10079994 (X-RAY DENTAL NORMAL) |
| 304 | Taste | >10001480 (AGEUSIA) |
| 305 | Taste | >10013911 (DYSGEUSIA) |
| 306 | Taste | >10020893 (HYPERTROPHY OF TONGUE PAPILLAE) |
| 307 | Taste | >10020989 (HYPOGEUSIA) |
| 308 | Taste | >10086446 (PRODUCT AFTER TASTE) |
| 309 | Taste | >10069227 (PRODUCT TASTE ABNORMAL) |
| 310 | Taste | >10082490 (TASTE DISORDER) |

**Figure S2.** Top Twenty Oral Adverse Events Reported After COVID-19 and Seasonal Influenza in the United States Stratified by Vaccine Group, January – December 2021 (CDC; VAERS-WONDER)

**
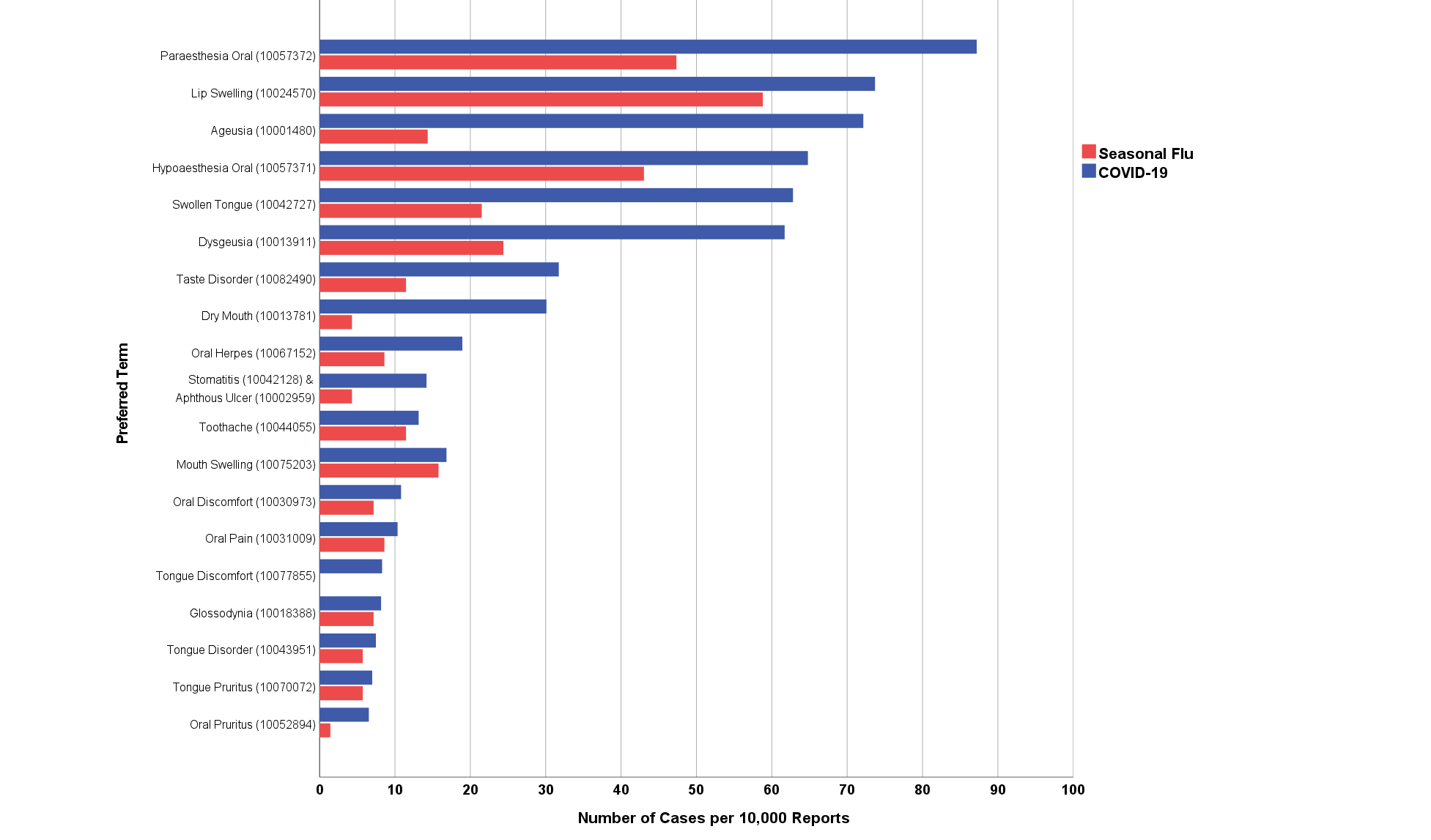
**
